# Biochemical Properties of Ectoine Hydroxylases from Extremophiles and Their Wider Taxonomic Distribution among Microorganisms

**DOI:** 10.1371/journal.pone.0093809

**Published:** 2014-04-08

**Authors:** Nils Widderich, Astrid Höppner, Marco Pittelkow, Johann Heider, Sander H. J. Smits, Erhard Bremer

**Affiliations:** 1 Laboratory for Microbiology, Department of Biology, Philipps-University Marburg, Marburg, Germany; 2 Max Planck Institute for Terrestrial Microbiology, Emeritus Group R.K. Thauer, Marburg, Germany; 3 X-Ray Facility and Crystal Farm, Heinrich-Heine-University Düsseldorf, Düsseldorf, Germany; 4 Institute of Biochemistry, Heinrich-Heine-University Düsseldorf, Düsseldorf, Germany; 5 LOEWE-Center for Synthetic Microbiology, Philipps-University Marburg, Marburg, Germany; Korea University, Republic of Korea

## Abstract

Ectoine and hydroxyectoine are well-recognized members of the compatible solutes and are widely employed by microorganisms as osmostress protectants. The EctABC enzymes catalyze the synthesis of ectoine from the precursor L-aspartate-β-semialdehyde. A subgroup of the ectoine producers can convert ectoine into 5-hydroxyectoine through a region-selective and stereospecific hydroxylation reaction. This compatible solute possesses stress-protective and function-preserving properties different from those of ectoine. Hydroxylation of ectoine is carried out by the EctD protein, a member of the non-heme-containing iron (II) and 2-oxoglutarate-dependent dioxygenase superfamily. We used the signature enzymes for ectoine (EctC) and hydroxyectoine (EctD) synthesis in database searches to assess the taxonomic distribution of potential ectoine and hydroxyectoine producers. Among 6428 microbial genomes inspected, 440 species are predicted to produce ectoine and of these, 272 are predicted to synthesize hydroxyectoine as well. Ectoine and hydroxyectoine genes are found almost exclusively in *Bacteria*. The genome context of the *ect* genes was explored to identify proteins that are functionally associated with the synthesis of ectoines; the specialized aspartokinase Ask_Ect and the regulatory protein EctR. This comprehensive *in silico* analysis was coupled with the biochemical characterization of ectoine hydroxylases from microorganisms that can colonize habitats with extremes in salinity (*Halomonas elongata*), pH (*Alkalilimnicola ehrlichii*, *Acidiphilium cryptum*), or temperature (*Sphingopyxis alaskensis*, *Paenibacillus lautus*) or that produce hydroxyectoine very efficiently over ectoine (*Pseudomonas stutzeri*). These six ectoine hydroxylases all possess similar kinetic parameters for their substrates but exhibit different temperature stabilities and differ in their tolerance to salts. We also report the crystal structure of the *Virgibacillus salexigens* EctD protein in its apo-form, thereby revealing that the iron-free structure exists already in a pre-set configuration to incorporate the iron catalyst. Collectively, our work defines the taxonomic distribution and salient biochemical properties of the ectoine hydroxylase protein family and contributes to the understanding of its structure.

## Introduction

The ability to sensitively detect and respond in a timely manner to changes in the external osmolarity through concerted genetic and physiological adaptation reactions is critical for the wellbeing and growth of most microorganisms [1], [2]. The accumulation of compatible solutes is a widely used strategy by members of both the *Bacteria* and the *Archaea* to offset the detrimental effects of high osmolarity on cellular hydration and physiology [3]–[5]. Compatible solutes are operationally defined as small organic osmolytes, highly water-soluble compounds whose physicochemical properties make them compliant with cellular biochemistry and physiology [6]–[9]. As a consequence, microbial cells can build-up compatible solute pools to exceedingly high intracellular levels, either through synthesis or uptake [1], [4], and they do this in a manner that is sensitively tied to the degree of the environmentally imposed osmotic stress [10], [11]. Accumulation of compatible solutes counteracts the efflux of water under hyperosmotic growth conditions; they thereby stabilize turgor and optimize the solvent properties of the cytoplasm [1], [6], [12]. These processes cooperate in strongly enhancing the growth of high osmolarity challenged cells.

Ectoine and its derivative 5-hydroxyectoine are well-recognized members of the compatible solutes [13], [14] and are effective osmostress protectants for microorganisms [15], [16]. Synthesis of ectoine proceeds from L-aspartate-β-semialdehyde and comprises three enzymatic steps that are catalyzed by L-2,4-diaminobutyrate transaminase (EctB), 2,4-diaminobutyrate acetyltransferase (EctA), and ectoine synthase (EctC) to yield the cyclic ectoine molecule [(4*S*)-2-methly-1,4,5,6-tetrahydropyrimidine-4-carboxylic acid] [17], [18]. The structural genes for the ectoine biosynthetic enzymes are typically organized in an operon (*ectABC*) [19] whose transcription is up-regulated in response to high osmolarity [11], [20]–[25]. Enhanced transcription of the *ect* genes is also triggered in some microorganisms by extremes in growth temperature [21], [26] as ectoines can also confer protection against both heat and cold stress [27]–[29]. A subgroup of the ectoine producers also synthesizes a hydroxylated derivative of ectoine, 5-hydroxyectoine [20], [30], in a biosynthetic reaction that is catalyzed by the ectoine hydroxylase (EctD) [20], [27], [31].

In addition to their role in alleviating osmotic stress, ectoines also serve as stabilizers of macromolecules and even entire cells [15], [32]. The function-preserving and anti-inflammatory effects of ectoines fostered substantial interest in exploring them for a variety of practical biotechnological applications and potential medical uses [15], [32]–[34].

Despite their closely related chemical structures, 5-hydroxyectoine often possesses superior stress protecting and function preserving properties than its precursor molecule ectoine [29], [35]–[38]. Here, we focus on the ectoine hydroxylase, the enzyme that forms (4*S*,5*S*)-2-methyl-5-hydroxy-1,4,5,6-tetrahydropyrimidine-4-carboxylic acid from the precursor ectoine through a region-selective and stereospecific hydroxylation reaction [13], [20]. The enzymatic characterization of the EctD protein from *Virgibacillus salexigens* [20] and *Streptomyces coelicolor* [29] identified the ectoine hydroxylase as a member of the non-heme-containing iron(II) and 2-oxoglutarate-dependent dioxygenase superfamily (EC1.14.11) [39]–[41]. The EctD-mediated hydroxylation of (4*S*)-ectoine to (4*S*,5*S*)-5-hydroxyectoine requires O_2_ and 2-oxoglutarate as co-substrates, thereby forming CO_2_, succinate, and 5-hydroxyectoine [20]. As seen in other members of the dioxygenase superfamily (e.g., the taurine dioxygenase TauD [42]), the EctD-catalyzed enzyme reaction is strictly dependent on a mononuclear ferrous iron center promoting the O_2_-dependent oxidative decarboxylation of 2-oxoglutarate, a sequence of events coupled with a two-electron oxidation of the substrate ectoine [43]. The high-resolution (1.85 Å) crystal structure of the *V. salexigens* EctD enzyme [44] revealed a protein fold that is commonly observed in members of the non-heme-containing iron(II) and 2-oxoglutarate-dependent dioxygenase superfamily, the so-called jelly-roll or cupin fold [40], [41]. The catalytically critical iron is coordinated by the side chains of a conserved H×D/E…H motive, the so-called 2-His-1-carboxylate facial triad [39]–[41].

To gain further insight into the properties of the ectoine hydroxylase and the taxonomic distribution of ectoine/hydroxyectoine producers, we have mined the genome sequences of members of the *Bacteria* and *Archaea* with fully sequenced genomes for the signature enzymes for ectoine (EctC) and hydroxyectoine (EctD) biosynthesis. We then explored the genome contexts of the *ect* gene clusters to identify those genes that are functionally associated with the production of ectoines, the specialized aspartokinase Ask_Ect [22], [45] or with the genetic control of *ect* gene expression, the repressor protein EctR [24], [25]. We coupled this comprehensive *in silico* analysis with the biochemical characterization of six EctD enzymes from phylogenetically widely separated bacteria covering various different lifestyles to define the properties and kinetic parameters of the ectoine hydroxylase on a broad basis. In addition, the crystal structure of the EctD protein from the salt tolerant moderate halophile *V. salexigens* in its iron-free form was solved, thereby allowing for the first time an assessment of the structural consequences of the binding of the active-site iron on the overall fold of the ectoine hydroxylase.

## Results and Discussion

### Database Searches for the Ectoine and Hydroxyectoine Biosynthetic Genes

**Figure 1:**
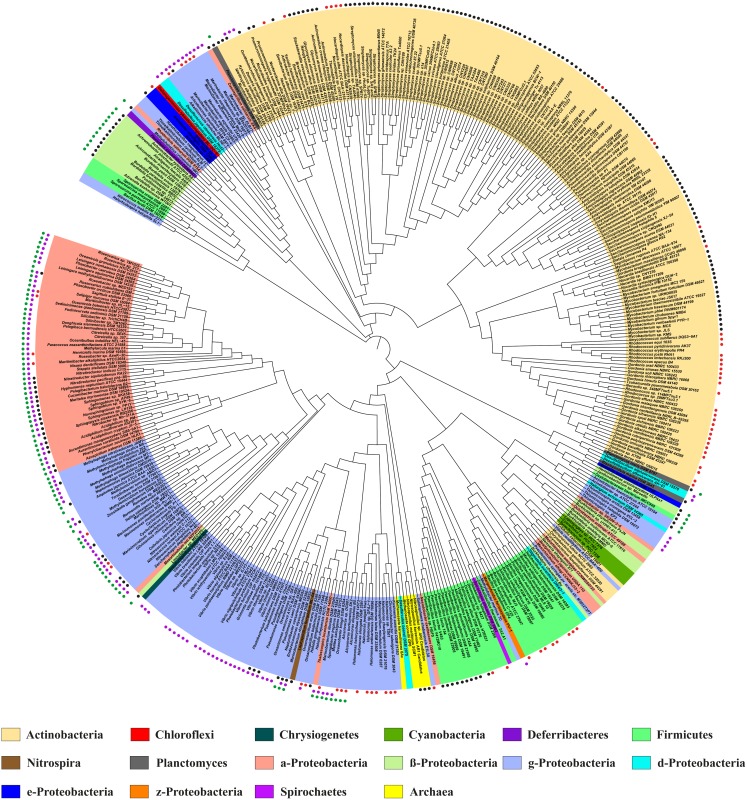
Phylogenetic tree of EctC- and EctD-type proteins. The shown phylogenetic tree is based on the alignment of EctC amino acid sequences identified by a BLAST search at the JGI Web-server that were then aligned using ClustalW. These compiled amino acid sequences were then used to assess the phylogenetic distribution of the EctC protein using the iTOL Web-server. Evolutionary distances are not given. The color code indicates the distribution of EctC among members of the *Bacteria* and *Archaea*. The presence of an *ectD* gene in a given microbial species possessing *ectC* is indicated by black (*ectD* is part of the *ect* gene cluster) or red circles (*ectD* is located outside of the *ect* gene cluster). Purple circles are indicating the presence of an *ask_ect* gene associated with the *ect* gene cluster, whereas the presence of an *ectR* regulatory gene is indicted by green circles. If different strains of the same species were sequenced, only one representative symbolizes them. For instance, there are genomic data of 139 strain of *Vibrio cholerae* available in the database, each of which possesses an *ectABC* gene cluster, but only one of these sequences was used for the phylogenetic analysis.

To assess the prevalence and taxonomic distribution of the ectoine and hydroxyectoine biosynthetic genes in microorganisms, we searched through finished microbial genome sequences at the database of the U.S. Department of Energy (DOE) Joint Genome Institute [46] for the presence of an *ectC* ortholog, coding for the signature enzyme of the ectoine biosynthetic pathway, the ectoine synthase [19]. As a search query for this database analysis, we used the amino acid sequence of the *V. salexigens* EctC protein (accession number: AAY29688) [20]. At the time of the database search, 6428 microbial genomes were represented that were derived from 6179 members of the *Bacteria* and 249 members of the *Archaea*. Of these genomes, 440 contained an *ectC* gene (approximately 7%), and most of them were members of the *Bacteria*; the notable exceptions were five *ectC* sequences present in the genomes of *Archaea* (two *Methanosaeta* and three *Nitrosopumilus* species). Excluding closely related strains of the same species for our analysis and using only a single representative, we constructed a phylogentic tree of the EctC sequences (Fig. 1). It is apparent from our database analysis that ectoine is a compatible solute which is synthesized almost exclusively by members of the *Bacteria* (Fig. 1). Genome sequences of 139 strains of *Vibrio cholerae* are represented among the 6428 searched microbial genomes, each of which is predicted to produce ectoine, but only one of them was included in the dataset depicted in Fig. 1. The few predicted archaeal ectoine producers have probably acquired the ectoine biosynthetic genes via lateral gene transfer events, since the exchange of genetic material between members of the kingdoms of the *Bacteria* and *Archaea* is a well-documented phenomenon [47].

We then assessed the distribution of the ectoine hydroxylase orthologs (*ectD*) in bacterial and archaeal genomes by using the *V. salexigens* EctD protein (accession number: AAY29689) [20] as the search query to identify those microorganisms predicted to produce hydroxyectoine. We found that 272 of the sequenced genomes possessed an *ectD* gene. Invariably these microorganisms also possessed an *ectC* gene, a result that is expected from the fact that hydroxyectoine is synthesized directly from the precursor molecule ectoine [20]. Hence, about two-thirds of the putative ectoine producers are predicted to synthesize hydroxyectoine as well (Fig. 1). As expected from the oxygen-dependent reaction of the EctD enzyme, *ectD* is never present in genomes of obligate anaerobes, although it is not universally present in aerobic or facultative species. Consistently, from the above mentioned archaeal ectoine-producing representatives, only the three (aerobic) *Nitrosopumilus* species possess an *ectD* gene as part of their *ect* gene clusters, whereas the genome sequences of the two (anaerobic) *Methanosaeta* species lacked *ectD* altogether.

### Overproduction and Purification of Recombinant Ectoine Hydroxylases from Extremophilic Bacteria

Biochemical properties of native ectoine hydroxylases from *V. salexigens* and *S. coelicolor* have been assessed previously [20], [29]. To determine whether the reported features of these two studied EctD proteins are representative for ectoine hydroxylases in general, we set out to study the characteristics of this type of enzyme on a broader basis. For these biochemical studies we chose six EctD proteins from the following taxonomically widely separated and mostly extremophilic microorganisms: *Halomonas elongata*, *Acidiphilium cryptum*, *Alkalilimnicola ehrlichii*, *Sphingopyxis alaskensis, Paenibacillus lautus*, and *Pseudomonas stutzeri*.

The Gammaproteobacterium *H. elongata* is the production strain for the industrial-scale manufacturing of ectoine [34] and grows in media with up to 5 M NaCl [48]. *A. cryptum* is an acidophilic metal-reducing Alphaproteobacterium that was isolated from an iron-rich sediment of an acid coal mine; it can grow at a pH of 5 [49], [50]. *A. ehrlichii* is an arsenite-oxidizing haloalkaliphilic Gammaproteobacterium isolated from Mono Lake (CA, USA) and has a pH optimum of 9.3 [51]. The Alphaproteobacterium *S. alaskensis* is a cold-adapted marine ultra-microbacterium that was isolated from permanently cold (4–10°C) water sources in the Resurrection Bay (AK, USA) [52], [53]. The Firmicute *P*. *lautus* was isolated from the Obsidian Hot spring in the Yellowstone National Park (WY, USA) that possesses a temperature range between 42–90°C; it can routinely be grown in the laboratory at 50°C [54]. The last studied microorganism was the nitrogen-fixing Gammabacterium *Pseudomonas stutzeri* strain A1501 that is not an extremophile, as it was isolated from plant roots [55]. Like the type strain of *P. stutzeri* (DSM 5109^T^), it produces 5-hydroxyectoine very efficiently and in preference over ectoine [22], [56], suggesting that its EctD enzyme might work particularly effectively.

**Figure 2:**
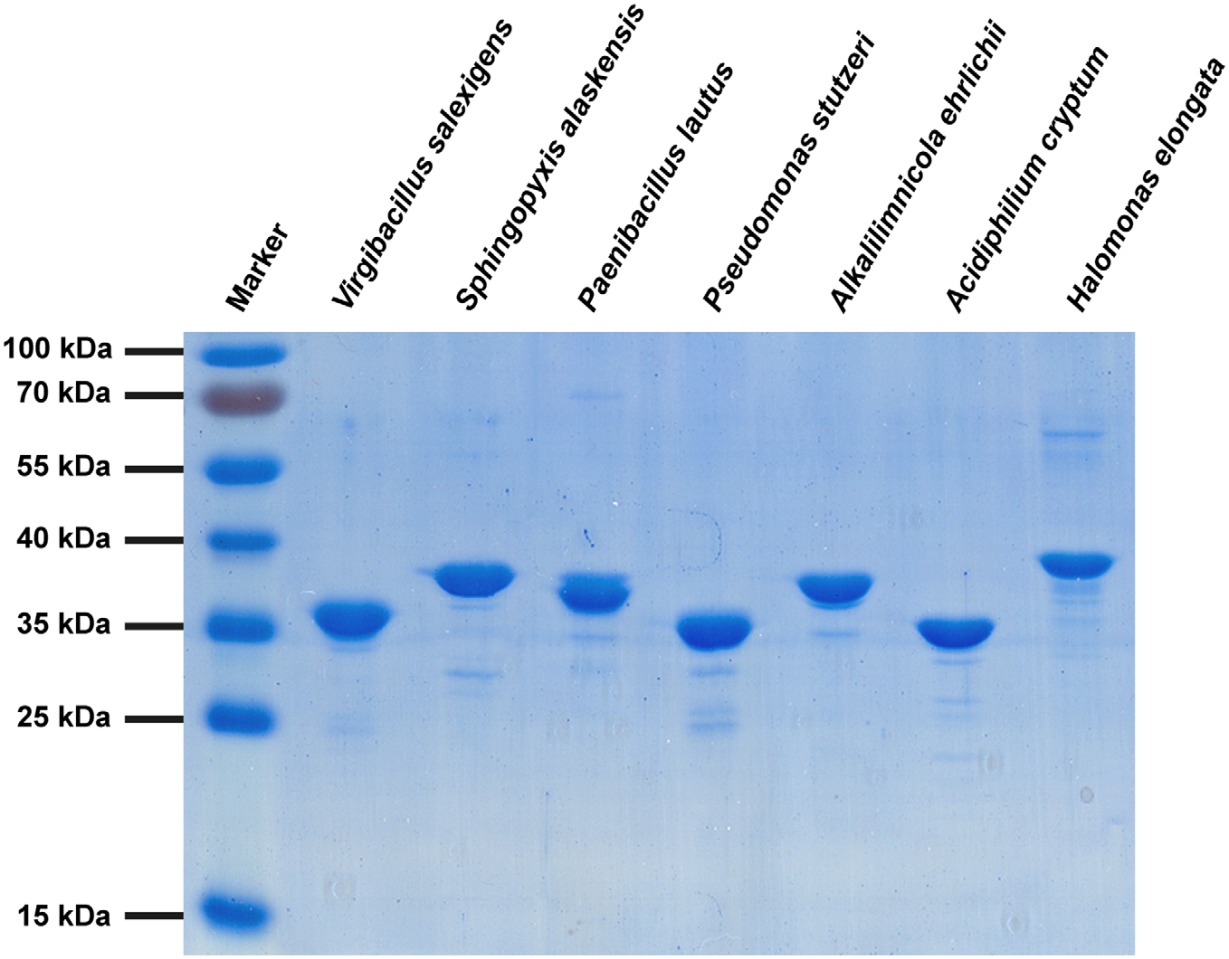
Purification of recombinant EctD proteins. A 12% SDS-PAGE of the recombinant EctD-type proteins originating from different microbial species after their overproduction in *E. coli* and purification via *Strep*-tag-II affinity chromatography is shown. 5 μg of each purified EctD protein were applied onto the gel. The gel was run at 25 mA for 2.5 h. The PageRuler Prestained Protein Ladder (Thermo Scientific, Schwerte, Germany) was used as marker. We note the presence of a overlapping second band in protein sample of the *Paenibacillus lautus* EctD preparation. This might stem from overloading the gel somewhat or from partial degradation of the purified EctD protein.

Given the very different habitats of these microorganisms, we wondered if the biochemical properties of their EctD proteins would reflect the preferences of their producers with respect to the salt, pH, and temperature parameters prevalent in their natural habitats. Using the biochemically and structurally well characterized *V. salexigens* EctD protein (*Vs*EctD) [20], [44] as a point of reference, the EctD proteins from the above-described six bacteria had an amino acid sequence identity ranging between 51% (*S. alaskensis*) and 40% (*H. elongata*). To study these EctD enzymes biochemically, we inserted the various *ectD* genes into an expression vector that allowed the production of the corresponding proteins as recombinant variants with a *Strep*-tag-II affinity peptide attached to their carboxy-terminus. These proteins could all be overproduced in an *Escherichia coli* host strain and isolated with good yields and purities by affinity chromatography on Step-Tactin Superflow material (Fig. 2). The amino acid sequences of the native EctD proteins range in length between 302 and 306 amino acids, except for EctD of *H. elongata,* which is predicted to consist of 332 amino acids (Table 1). The migration of some of the purified recombinant EctD proteins on a 12% SDS-polyacrylamide gel (Fig. 2) deviates somewhat from their calculated molecular mass (Table 1), a property that might be connected with the particular amino acid composition of individual EctD proteins.

**Table 1:** Biochemical properties of the studied EctD-type proteins.

EctD from	length[AS]	mass[kDa]	pI	optimumtemp. [°C]	temp.range [°C]	pH	pH range	optimumKCl [mM]	KClrange [mM]	optimumNaCl [mM]	NaClrange [mM]
*V. salexigens*	300	34.4	5.8	32	5–50	7.5	5.5–9.6	150	0–750	100	0–350
*S. alaskensis*	306	34.1	5.5	40	5–50	8.0	5.5–9.6	100	0–1000	100	0–500
*H. elongata*	332	37.4	5.8	32	5–47	8.0	6.5–9.6	150	0–750	100	0–250
*P. stutzeri*	302	34.2	5.5	35	10–50	7.5	5.5–9.6	150	0–1000	100	0–350
*P. lautus*	302	34.8	5.6	40	15–50	7.5	5.5–9.6	200	0–750	150	0–250
*A. ehrlichii*	302	34.3	5.7	35	15–45	7.5	6.5–9.6	150	0–1000	150	0–400
*A. cryptum*	306	34.1	5.8	32	10–47	8.0	5.5–9.6	100	0–1000	50	0–300

The biochemical properties of the studied EctD-type proteins were determined as described in Material and Methods. The given temperature, pH and salt ranges describe a window in which the tested enzymes still exhibited some degree of activity.

Since the presence of a correctly complexed iron ligand is critical for EctD-mediated enzyme catalysis [20], [43], [44], we determined the iron-content of each of these recombinant proteins and found between 0.87 and 0.96 mole iron per mol of EctD protein. Hence, these recombinant EctD proteins should all be functional. An initial assessment of their enzymatic activities under the same assay conditions as used previously for the ectoine hydroxylases from *V. salexigens* and *S. coelicolor* [20], [29] demonstrated that this was indeed the case.

### Biochemical Properties of the Ectoine Hydroxylases

**Figure 3:**
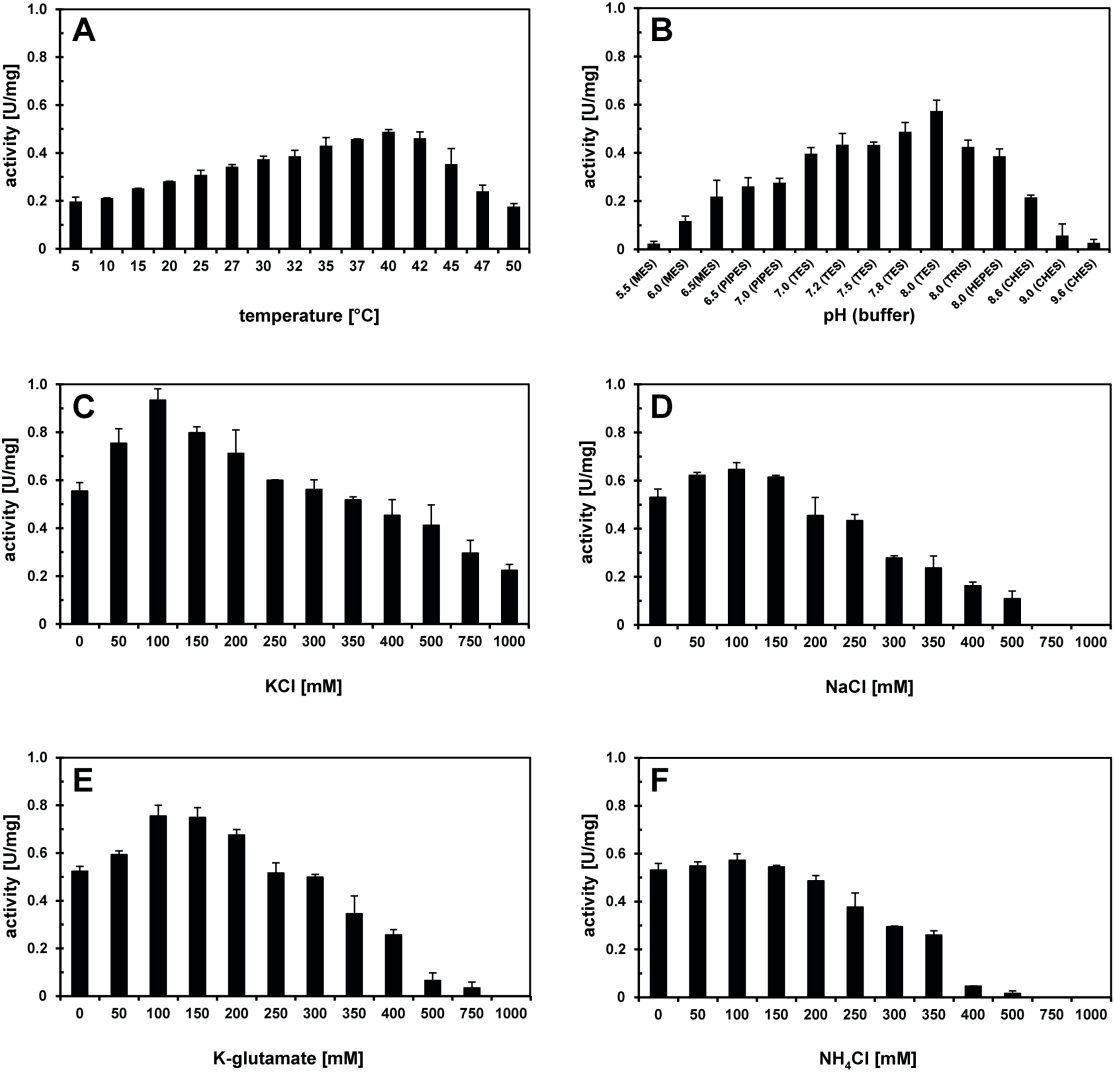
Biochemical properties of the EctD enzyme from *S. alaskensis*. The enzyme activity of the ectoine hydroxylase from *S. alaskensis* is shown with respect to (A) the temperature optimum, (B) the pH optimum and the influence of different salts: (C) potassium chloride, (D) sodium chloride, (E) potassium glutamate and (F) ammonium chloride.

We determined for each of the EctD enzymes its temperature and pH optimum and measured the influence of various salts (KCl, NaCl, K-glutamate, NH_4_Cl) on the catalytic efficiency. The data from this set of experiments are summarized in Table 1 and are documented in detail for the *S. alaskensis* enzyme in Fig. 3. The data for all other enzymes are summarized in Fig. S1 to Fig. S5. Overall, the basic biochemical parameters of the six newly studied EctD enzymes and the re-analyzed EctD protein from *V. salexigens* [20] were all quite similar (Table 1), regardless of the environmental parameters that were prevalent in the habitats of those microorganisms from which they originate. However, differences were noted with respect to their resistance to the inhibiting action of increased salt concentrations (Table 1).

In studying the biochemical properties of the ectoine biosynthetic enzymes from *H. elongata*, Ono *et al*. [17] reported that the *in vitro* activity of these proteins was strongly dependent on high concentrations of NaCl (0.4–0.5 M), a type of salt that is unlikely to be accumulated to such high levels *in vivo* by osmotically stressed *H. elongata* cells, since sodium ions are toxic for bacterial cells. We did not find any strong stimulating effect of high NaCl concentrations on any of the ectoine hydroxylases we studied here (Table 1), including that of *H. elongata* (Fig. S1). On the contrary, high concentrations of NaCl typically inhibited the enzyme activities of the EctD variants (Fig. 3 and Fig. S1 to Fig. S5). However, notable stimulating effects [about two- to three-fold (Fig. 3 and Fig. S1 to Fig. S5)] on EctD enzyme activities were recorded with KCl or K-glutamate solutions.

We assessed the quaternary structure of the six newly studied EctD proteins by gel filtration. An example of this analysis is shown in Fig. S6 for the *S. alaskensis* EctD protein. The protein eluted between 72 to 83 ml (maximum: 77. 5 ml) from the size exclusion chromatography column and thereby corresponds to a protein of about 70.4 kDa. Since the calculated molecular mass of the *S. alaskensis* EctD protein monomer with the attached *Strep-*tag-II affinity peptide (nine amino acids) is 35.29 kDa, the ectoine hydroxylase is apparently a homodimer. The same conclusion was derived for all other analyzed EctD proteins (data not shown), including that from *V. salexigens*, which has previously been suggested to be a monomer [20].

### Temperature Stability of the Ectoine Hydroxylases: The *S. Alaskensis* and *P. Lautus* Enzymes Stand Out

**Figure 4:**
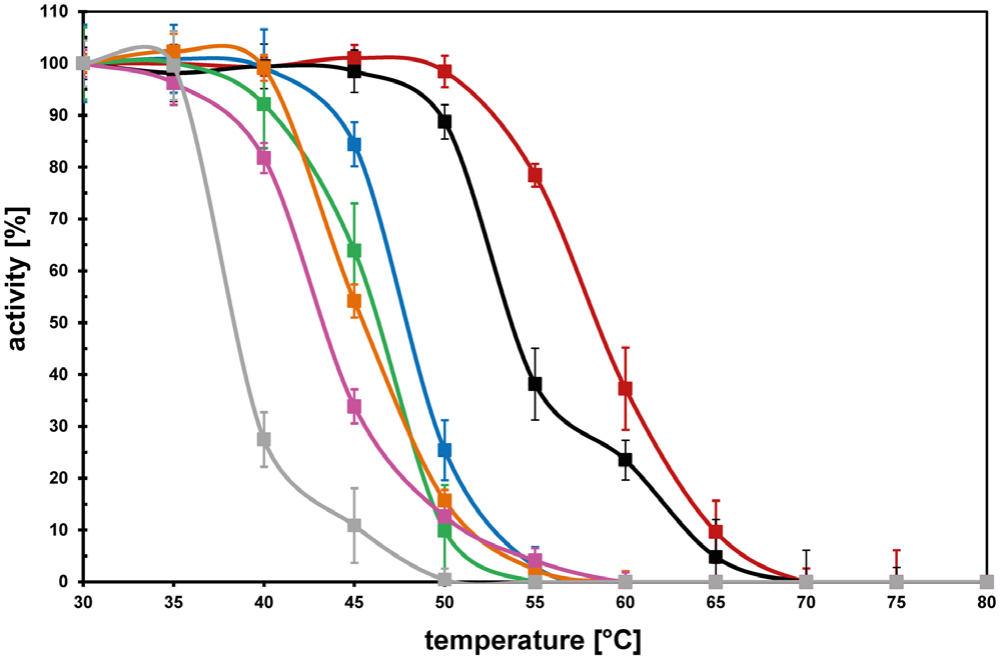
Resistance of various ectoine hydroxylases against the denaturing effects of high temperature. The temperature profiles of the ectoine hydroxylases from *H. elongata* (grey), *A. cryptum* (pink), *A. ehrlichii* (orange), *V. salexigens* (green), *P. stutzeri* (blue), *S. alaskensis* (black), and *P. lautus* (red) are given. Each EctD protein was pre-incubated at the indicated temperatures for 15 min before its specific activity was then determined under its optimal assay condition. The enzyme activity exhibited by each enzyme after pre-incubation at 30°C was set as 100%.

The studied EctD enzymes have similar temperature optima but differ in the range of temperatures in which they operate naturally (Table 1). To investigate this further, we studied their temperature stability. For these experiments, we pre-incubated 100 μg of each enzyme in 100 μl TES-buffer (pH 7–8) for 15 min at a given temperature and then measured its activity under assay and temperature conditions that had been optimized for each individual EctD protein (Table 1). The ectoine hydroxylase from *H. elongata* turned out to be the most temperature labile protein, whereas those from *S. alaskensis* and *P. lautus* proved to be quite temperature resistant; all other enzymes possessed intermediate degrees of temperature stability (Fig. 4). The strong temperature resistance of the *P. lautus* EctD protein does not come as a surprise since this *Paenibacillus* species was isolated from a hot spring with water temperatures ranging between 42–90°C [54]. The considerable heat tolerance of the *S. alaskensis* EctD enzyme is more of a surprise since this bacterium is well adapted to permanently cold (4–10°C) marine environments although it can grow at higher temperatures [52].

### Kinetic Parameters of Ectoine Hydroxylases

After having optimized the parameters of the enzyme activity assays for each of the six purified ectoine hydroxylases (Table 1), we determined their apparent kinetic parameters for the co-substrate 2-oxoglutarate and the substrate ectoine (Table 2). This assessment showed that the studied ectoine hydroxylases all possess similar kinetic parameters. For instance, the *S. alaskensis* enzyme had an apparent *K_m_* of 9.8±0.5 mM for its substrate ectoine and of 2.7±0.3 mM for its co-substrate 2-oxoglutarate, respectively, a *V_max_* of 1.0±0.2 U mg^−1^, a *k_cat_* of 1.2 s^−1^ per holoenzyme and a catalytic efficiency of 0.12 mM^−1 ^s^−1^ (Table 2). The *P. stutzeri* and *V. salexigens* enzymes stand out among the tested enzymes with respect to their catalytic efficiencies with values of 1.44 mM^−1 ^s^−1^ and 1.31 mM^−1 ^s^−1^, respectively (Table 2). In contrast, the EctD enzyme from the industrially used ectoine/hydroxyectoine production strain *H. elongata* [32], [34] exhibits no particularly notable features with respect to its catalytic efficiency (0.49 mM^−1 ^s^−1^) (Table 2). The relatively good performance of the ectoine hydroxylase from *P. stutzeri* A1501 is certainly consistent with the preferred accumulation of 5-hydroxyectoine by osmotically stressed cells of this isolate over that of ectoine [22], a feature that is also found in the type strain (DSM 5190^T^) of *P. stutzeri* [56].

**Table 2:** Kinetic parameters of the analyzed ectoine hydroxylases.

EctD from	*K_m_* [mM ectoine]	*v_max_* [U/mg]	*k_cat_* [s^−1^]	*k_cat_*/*K_m_* [mM^−1 ^s^−1^]	*K_m_* [mM 2-oxoglutarate]
*V. salexigens*	5.9±0.3	6.4±0.2	7.7	1.31	4.9±0.3
*S. alaskensis*	9.8±0.5	1.0±0.2	1.2	0.12	2.7±0.3
*H. elongata*	5.7±0.6	2.5±0.2	2.8	0.49	4.8±0.4
*P. stutzeri*	6.2±0.4	6.7±0.2	8.9	1.44	4.6±0.5
*P. lautus*	9.5±0.7	1.3±0.1	1.6	0.17	3.9±0.2
*A. ehrlichii*	9.0±0.3	1.0±0.1	1.2	0.13	5.0±0.3
*A. cryptum*	10.0±0.6	2.8±0.3	3.4	0.34	4.1±0.4

The kinetic parameters of the studied EctD enzymes were determined under conditions that were optimal for each enzyme (see Table 1) by independently varying the substrate concentration of ectoine between 0 and 40 mM and that of the co-substrate 2-oxoglutarate between 0 and 50 mM. The *k_cat_* values were determined per holoenzyme (a homo-dimer of the EctD protein) and the catalytic efficiency for the hydroxylation of ectoine is given as *k_cat_*/*K_m_*.

Our data show that ectoine hydroxylases are not particularly effective enzymes since their affinities for their substrate ectoine and their co-substrate 2-oxoglutarate are low with apparent *K*_m_ values in the mM range, and they exhibit only modest *V_max_* numbers and catalytic efficiencies (Table 2). These properties of EctD enzymes have also been observed previously when the ectoine hydroxylases from *V. salexigens*, and *S. coelicolor* were isolated as native proteins from their natural producer bacteria and not as recombinant proteins as done here [20], [29]. The moderate kinetic parameters of the ectoine hydroxylase might be connected to the fact that in osmotically stressed microbial cells, ectoine is typically accumulated first and 5-hydroxyectoine production then sets in only after a substantial cellular pool of its precursor molecule has been built up [22], [29], [57]. Furthermore, the *in vitro* activities of the EctD enzymes require considerable 2-oxoglutarate concentrations in order to work efficiently (Table 2) [20], [29], and therefore the cellular 2-oxoglutarate pool [58] could potentially limit 5-hydroxyectoine formation *in vivo*.

### EctD Enzyme Activity is not Reversible

In their recent excellent overview on the role of ectoines as microbial stress protectants and their biotechnological applications, Pastor *et al*. [15] suggested that the EctD enzyme may also catalyze the reverse reaction to form ectoine from 5-hydroxyectoine (see Fig. 2 in [15]), albeit without providing any experimental evidence or presenting a possible mechanism. We therefore assayed for the stability of the products of the enzyme reaction of the *V. salexigens* EctD protein under conditions set up to favor a hypothetical backward reaction (6 mM 5-hydroxyectoine, 10 mM bicarbonate, and 20 mM succinate as potential substrates were incubated with 40 μg EctD protein) and found no decrease of the hydroxyectoine concentration or production of any ectoine, even after incubating the enzyme reaction mixture for 24 hours (Fig. S7). For comparison, 6 mM ectoine are almost completely converted into 5-hydroxyectoine within 20 min when 10 mM 2-oxoglutarate were provided as the co-substrate (Fig. S7). This is also predicted from the highly exergonic thermodynamics of ectoine hydroxylation by EctD (estimated ΔG°’ < −400 kJ/mol), which should completely preclude the backward enzyme reaction [39]–[41], [59]. We therefore conclude that the ectoine hydroxylase is an enzyme that operates exclusively in one direction under physiologically relevant conditions to direct the formation of 5-hydroxyectoine from the precursor ectoine.

### Crystal Structure of the *V. Salexigens* EctD Protein in its Iron-free Form

A high-resolution (1.85Å) crystal structure of the EctD protein from *V. salexigens* has previously been reported in complex with the catalytically important iron ligand; however, it lacks the co-substrate 2-oxoglutarate and the substrate ectoine [44]. This structure was recently used as a starting point for molecular dynamics simulations and site-directed mutagenesis experiments to glean information about the coordination of the ligands within the EctD active site [43]. We continued our efforts to obtain an EctD crystal structure containing all ligands and therefore pushed the recombinant production of the *V. salexigens* EctD protein in *E. coli* to very high levels in order to supply the large quantities of protein needed for the crystallization trials. In this way, we increased the amounts of the purified *V. salexigens* recombinant EctD enzyme from about 20–25 mg per liter of culture (the ectoine hydroxylase source for biochemical studies) to 200–300 mg per liter of culture. However, after analyzing the iron content of this strongly overproduced EctD enzyme preparation, it became apparent that most of the isolated proteins did not contain an iron molecule; the iron content of the EctD protein solution dropped to 0.1–0.2 mole iron per 1 mol of EctD, rendering the enzyme largely inactive. Upon addition of Fe^2+^ ions prior to the enzyme activity measurements, we observed that the activity returned to levels observed before [20], [43], indicating that the missing iron catalyst in the purified EctD protein can be restored after the protein has adopted its native cupin barrel fold [44].

**Figure 5:**
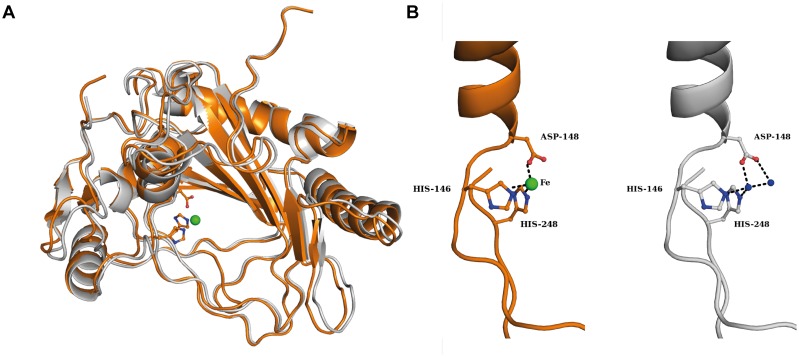
Crystal structure of the apo-form of the ectoine hydroxylase from *V. salexigens*. (A) Overlay of the crystal structure of the apo-EctD protein (colored in grey) with the Fe-bound crystal structure of EctD (colored in orange) in cartoon representation. The Fe ion of the Fe-bound EctD protein is represented as a green sphere. Data coordinates for the iron-bound form of the *V. salexigens* EctD protein were taken from the protein database (PDB) entry 3EMR and those from the iron-free form were from PDB entry 4NMI. (B) Details of the molecular determinants of the iron-binding site of the *V. salexigens* EctD protein in its iron bound (orange) and iron-free (grey) forms. The side chains of the iron-binding residues Asp148, His146 and His248 are highlighted. Green and blue spheres represent the bound iron and water molecules, respectively.

These observations prompted us to explore whether the *V. salexigens* EctD protein adopts a similar conformation in its iron-free and iron-bound forms, or whether the incorporation of the iron ligand leads to substantial structural changes. We grew EctD crystals and collected a 1.9 Å X-ray dataset. The cell constants and the space-group (Table S1) were identical to the structure of the iron-bound EctD, suggesting that the apo-EctD protein crystalized in a manner similar to that found in the iron bound form [44]. After solving the new crystal structure of the EctD protein, it became apparent that the iron ligand was lacking, as evidenced by the missing pronounced electron density that is present in the iron-bound EctD crystal structure [44]. Otherwise, the apo- and the iron-bound forms are almost identical, as indicated by the RMSD value of 0.34 Å over 280 Cα atoms. An overlay of both EctD crystal structures is shown in Fig. 5A.

In the *V. salexigens* EctD protein, the iron ligand is bound via interaction with two histidine side-chains, His-146 and His-248, and the side-chain of Asp-148 (Fig. 5A and B) [44]. Together these residues form a conserved H×D/E…H motif, the so-called 2-His-1-carboxylate facial triad. [39]–[41], [59]. A comparison of the iron-binding residues in the apo- and iron-bound structures of the *Vs*EctD protein shows that they exhibit the same architecture, except that the iron ligand is present in one structure and absent in the other (Fig. 5B). Interestingly, in the apo-structure of EctD, two water molecules populate the iron-binding site formed by the 2-His-1-carboxylate facial triad. This keeps the side chains of the His-146, His-248 and Asp-148 in an orientation very similar to that observed in the iron-bound EctD crystal structure (Fig. 5B). Hence, the EctD apo-protein exists in a form that is pre-set to incorporate the iron catalyst [43].

### Phylogenetic Distribution of the *EctC* and *EctD* Genes

Previous studies have indicated that the ability to produce ectoine and hydroxyectoine is widely distributed in the microbial world but is absent from eukarya [15], [34], [44], [60]. We updated and extended this information on a genome-wide scale in the following way: (i) first, we visualized the relationship among the 440 retrieved EctC sequences via the iTOL tool [61] to analyze their taxonomic association with members of the *Bacteria* and *Archaea*; (ii) we then projected the information on the presence of the ectoine hydroxylase in a given microbial species onto this phylogenetic tree of the EctC protein to reveal the extent and taxonomic distribution of EctD protein among putative ectoine producers; (iii) we inspected the genome context of each of the 440 microbial species that possessed *ectC* and from this bioinformatics approach retrieved the genetic organization of the *ect* biosynthetic gene cluster; (iv) furthermore, we assessed the co-localization of the *ect* genes with genes that have been functionally associated with ectoine/hydroxyectoine biosynthesis, the gene for a specialized aspartokinase Ask_Ect [22], [45], [60], and that of the transcriptional regulator EctR [24], [25].

In the first step of this *in silico* analysis, we aligned the retrieved 440 EctC sequences using the ClustalW [62] algorithm and found amino acid sequence identities that ranged between 88% and 27% with reference to the *V. salexigens* EctC protein [20]. The corresponding numbers for the degree of identity of the 272 EctD proteins range between 79% and 37% with reference to the *V. salexigens* EctD protein. The visualization of the taxonomic distribution of the EctC and EctD proteins with the iTOL-software package [61] revealed putative bacterial and archaeal ectoine producers in 17 phyla (Fig. 1). Fifteen of these phyla are taxonomically associated with the domain of the *Bacteria* and two with the domain of the *Archaea*. The taxonomic distribution of the putative hydroxyectoine producers was more restricted: ectoine hydroxylase genes are found only in nine phyla (Fig. S8). In the following, we further consider the ectoine synthase, the ectoine hydroxylase, the specialized aspartokinase Ask_Ect, and the transcriptional regulator EctR.

### The Ectoine Synthase EctC

The ectoine biosynthetic enzymes L-2,4-diaminobutyrate transaminase (EctB; EC 2.6.1.76) and the 2,4-diaminobutyrate acetyltransferase (EctA; EC 2.3.1.178) have isoenzyme counterparts in various biochemical pathways [17], [18], but the ectoine synthase (EctC; EC 4.2.1.108) is unique. Therefore, EctC has been considered so far as the diagnostic enzyme for ectoine production. It catalyzes the cyclization of *N*-γ-acetyl-2,4-diaminobutyrate to ectoine via a water elimination reaction [17], [18], whereas *N*-α-acetyl-2,4-diaminobutyrate that is formed during ectoine catabolism [34] is apparently no substrate for this enzyme. In a reversal of the native cyclization reaction scheme, EctC can also inefficiently hydrolyze synthetic ectoine derivatives with reduced or expanded ring sizes and can catalyze the cyclic condensation of glutamine to the synthetic compatible solute 5-amino-3,4-dihydro-2H-pyrrole-2-carboxylate (ADPC) as a side reaction [57].

The distribution of the ability to synthesize ectoine, as indicated by the presence of an *ectC* gene in a given genome sequence, appears to extend mostly to members of the Proteobacteria, Firmicutes, or Actinobacteria (Fig. 1). Moreover, the EctC sequences cluster in large part with the taxonomic subgroups of these bacterial phyla and their branching order occurs in parallel with the different corresponding taxonomic units of their microbial hosts down to the order level. This suggests a long co-evolution of the ectoine synthesis genes in the various bacteria. Moreover, the analysis revealed a conspicuous abundance of marine species among the putative proteobacterial ectoine producers. On the other hand, the ectoine-producing species of the Firmicutes and Actinobacteria are mostly from terrestrial habitats. The few EctC sequences from species with different taxonomic affiliations are interspersed into large clusters of related species (Fig. 1) and can probably be explained by lateral gene transfer events. Thirteen amino acid residues were fully conserved among all of the 440 inspected EctC proteins, but in contrast to EctD [20], [44], no signature sequence of the ectoine synthase was readily discernable.

**Figure 6:**
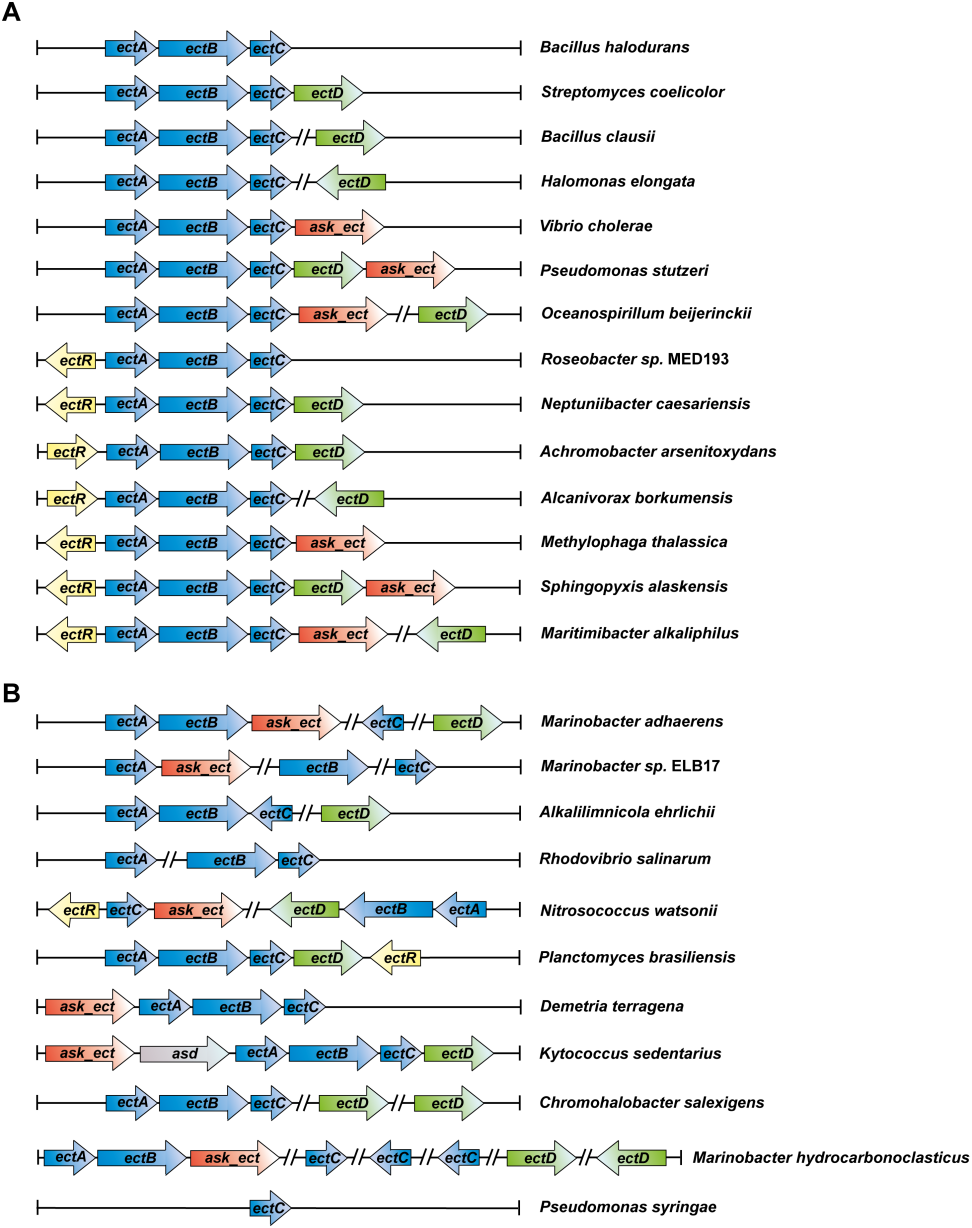
Genetic organization of the ectoine/hydroxyectoine biosynthesis gene clusters. The different types of *ect* gene clusters present in putative ectoine/hydroxyectoine producers are represented. An example for the genetic organization of each type of *ect* cluster found in the *ectC* reference set (440 representatives; Fig. 1) is given along with a microorganism in which it occurs. (A) Most common organizational types of the *ect* gene clusters. (B) Representatives of the organization of the ectoine/hydroxyectoine biosynthetic genes that deviate from the otherwise commonly found genetic organization.

In our dataset of 440 putative ectoine producers, we found 22 organisms containing solitary genes for EctC-type proteins that were not associated with the characteristic *ectA* and *ectB* ectoine biosynthetic genes [19] (Fig. 6). They all possess genes for EctB-related proteins somewhere else in their genome, but none of them possess genes for recognizable EctA-like proteins. Notably, the *ectD* gene is also completely absent from this group of bacteria (Fig. 1). Kurz and co-workers [63] investigated the ectoine biosynthetic potential of the plant pathogen *Pseudomonas syringae* pv. *syingae* B728a, a strain with such an orphan *ectC* gene, and found that it produced ectoine only under osmotic stress conditions when surface-sterilized leaves of the host plant *Syringa vulgaris* were added to the bacterial culture. Furthermore, in functional complementation experiments, the corresponding ectoine synthase was only partially active and, surprisingly, feeding of the direct ectoine precursor, *N*-γ-acetyl-2,4-diaminobutyrate, to the *P. syringae* pv. *syingae* B728a did not lead to ectoine synthesis although this compound was taken up by the cells [63].

Inspection of the EctC phylogenetic tree (Fig. 1) showed that the 22 host species possessing these solitary *ectC* genes are taxonomically rather diverse. The retrieved amino acid sequences are all phylogenetically related to a cluster of EctC proteins present primarily in members of the Firmicutes that all possess intact ectoine biosynthetic pathways and that are predicted to produce both ectoine and hydroxyectoine (Fig. 1). In our view, the functional relationships of these solitary *ectC* genes cannot yet be fully determined with confidence: (i) the species possessing orphan EctC-type proteins may be actual ectoine producers that have to rely on an environmental supply of ectoine precursor molecules as suggested by the data reported by Kurz *et al*. [63]; (ii) these EctC-like proteins may be evolutionary remnants of a previously intact ectoine biosynthetic pathway; or (iii) may have evolved (or be in the process of evolving) towards biochemical activities other than the cyclization of the direct ectoine precursor molecule *N*-γ-acetyl-2,4-diaminobutyrate.

### The Ectoine Hydroxylase EctD

The ectoine hydroxylase [20], [27], [31] is frequently confused in genome annotations with proline- or phytanoyl-hydroxylases that, like EctD, also belong to the non-heme-containing iron(II) and 2-oxoglutarate-dependent dioxygenase superfamily (EC1.14.11) [39]–[41]. However, *bona-fide* EctD-type proteins can be distinguished from the latter two enzymes by the presence of a strictly conserved signature sequence [20], [44]. This stretch of 17-amino acids [Phe-143 to Pro-159 in the *Vs*EctD protein] not only serves an important role for the structuring of the overall fold of the EctD cupin barrel [44], but it also contains a number of residues implicated by *in silico* modeling and by structural and mutational analysis in the binding of iron, 2-oxoglutarate and ectoine [43].

*ectD* genes are only present in a subset of strains that also possess the full set of ectoine biosynthetic genes but the extent of *ectD* occurrence varies widely between different bacterial taxa. The *ectD* gene can either be part of the *ectABC* operon or can be encoded somewhere else in the genome [20], [27], [31] (Fig. 6). Of the putative 272 hydroxyectoine producers, 72% possess *ectD* genes that are located next to the *ectABC* gene cluster (Fig. 1 and Fig. 6). As expected from the oxygen dependence of the ectoine hydroxylase enzyme reaction [20], [43], none of the obligately anaerobic ectoine-producing bacterial or archaeal species contains an *ectD* gene. The highest incidence of *ectD* is observed in the actinobacterial ectoine producers, in particular in all sequenced species of the genera *Streptomyces* and *Actinomadura,* all fast-growing (but none on the slow-growing) species of *Mycobacterium,* and most sequenced species of the orders *Pseudonocardiales*, *Glycomycetales*, the genus *Nocardiopsis* and the phylogenetically basal genus *Nitriliruptor* (Fig. 1 and Fig. S8). It is noteworthy in this context that the ectoine hydroxylase from *S. coelicolor* has been biochemically characterized [29] and it exhibits kinetic parameters similar to the six ectoine hydroxylases that were functionally assessed in this study (Table 2). There are only three species belonging to the *Archaea* that are predicted to synthesize hydroxyectoine. They are all members of the aerobic thaumarchaeal genus *Nitrosopumilus* [64], and they share very similar *ectD* gene products with those of the gammaproteobacterial genus *Nitrosococcus*. As both genera represent marine nitrifying microorganisms, recent gene sharing by lateral gene transfer [47] seems quite plausible.

### The Specialized Aspartokinase Ask_Ect

Ectoine is formed from L-aspartate-β-semialdehyde [17], [18], a central hub in microbial amino acid metabolism, cell wall biosynthesis, and antibiotic production [65]. L-aspartate-β-semialdehyde is synthesized from L-aspartate through the subsequent enzymatic activities of the aspartokinase (Ask) and the aspartate-semialdehyde-dehydrogenase (Asd). Complex transcriptional and posttranscriptional control mechanisms directed towards the expression of the *ask* gene and the enzymatic activity of its encoded protein ensure that there is no over- or undersupply of L-aspartate-β-semialdehyde [65].

The cellular pool of L-aspartate-β-semialdehyde is a potential bottleneck for the massive ectoine synthesis setting in under high osmolarity growth conditions [66]. To avoid such a metabolic constraint, a sub-group of the ectoine producers increase the cellular level of a specialized aspartokinase, Ask_Ect, simultaneously with the amounts of the EctABC biosynthetic enzymes through the co-transcription of the corresponding structural gene (*ask_ect*) with the *ectABC/D* gene cluster [22], [45]. However, it is apparent from our database analysis that the majority of the ectoine/hydroxyectoine producers possibly circumvent such an anabolic bottleneck without producing an aspartokinase that is specifically earmarked for ectoine production. We found that about 30% (132 microbial species) of the predicted 440 ectoine/hydroxyectoine producers possess an *ask_ect* gene. This gene is almost exclusively found in Alpha-, Gamma- and Deltaproteobacteria; the taxonomic distribution among the Gammaproteobacteria is somewhat patchy but it is quite regular in the other two subphyla and the gene seems to be absent in Betaproteobacteria (Fig. 1). Strikingly, none of the predicted ectoine/hydroxyectoine producers that belong either to the Firmicutes or to the Actinobacteria (except three species) possess an *ask_ect* gene in the vicinity of their *ectABC/D* gene clusters (Fig. 1).

Aspartokinases are ubiquitously found in microorganisms and several aspartokinases with distinct regulatory features are often present in the same bacterial cell [65]. The latter is true for the ectoine/hydroxyectoine producer *Pseudomonas stutzeri* A1501, where a comparative biochemical analysis of the specialized aspartokinase Ask_Ect and the anabolic aspartokinase LysC revealed distinct feedback inhibition profiles by metabolites [22].

### The Transcriptional Regulator EctR

The functional association of the *ectR* gene with ectoine biosynthesis was first demonstrated in the halotolerant methanotrophic Gammaproteobacterium *Methylomicrobium alcaliphilum*, where EctR serves as a repressor of *ectABC-ask_ect* gene transcription [25]. Notably, the elevated transcription of the ectoine biosynthetic genes in an *ectR* mutant of *M. alcaliphilum* remains osmotically inducible [25]. It is currently not known which environmental or cellular cues dictate the binding to or the release of EctR from its operator sequence. In *M. alcaliphilum*, the EctR operator overlaps the −10 sequence of the *ect* promoter and EctR might also regulate the expression of its own structural gene [25]; however, this latter regulatory feature does not always seem to exist [24]. The EctR repressor protein is a member of the widely distributed group of MarR transcriptional regulators but forms a distinct sub-group within this superfamily [24]. Of the 440 putative ectoine/hydroxyectoine producers, 24% possess *ectR*-type genes (107 microbial species) (Fig. 1) whose transcriptional direction is frequently oriented divergently from that of the *ect* gene cluster (Fig. 6). EctR is found almost exclusively among the Proteobacteria; all Alpha- and Betaproteobacteria that are predicted to synthesize ectoines possess an *ectR* gene, whereas its distribution among the Gammaproteobacteria is more irregular (Fig. 1).

It is worth noting that in *Vibrio cholerae* an EctR-related MarR-type transcriptional regulator (CosR) has been described that negatively controls ectoine biosynthetic and compatible solute uptake genes in response to the ionic strength of the growth medium. CosR from *V. cholerae* and EctR from *M. alcaliphilum* exhibit 51% amino acid sequence identity; however, unlike *ectR*, the *cosR* gene is not located in the vicinity of the ectoine gene cluster present in *V. cholerae* [67]. Ectoine biosynthesis, but not that of hydroxyectoine, is widespread among *V. cholerae* strains [68] and other *Vibrio* species [69], microorganisms that primarily live in marine habitats and estuarine ecosystems. For instance, genome sequence of 139 *V. cholerae* strains have been deposited in the databases and each of these strains is predicted to synthesize ectoine (data not shown).

### Genetic Organization of the Ectoine and Hydroxyectoine Structural Genes

After the initial discovery of the *ectABC* gene cluster for the synthesis of ectoine in *Marinococcus halophilus* [19], transcriptional profiling of the corresponding genes in several Gram-negative and Gram-positive bacteria showed that they were transcribed as operons inducible by osmotic or temperature stress [11], [20], [21], [23], [26], [27]. In some ectoine producers, the *ectABC* genes are expressed from a single promoter [11], [20], whereas in others a more complex pattern of regulation of this gene cluster has been reported [21], [24], [34]. Evidence for the presence of a putatively nitrogen-responsive Sig-54 type promoter driving the separate expression of *ectC* has been presented in the case of *H. elongata* [34].

We inspected the genetic organization of the *ect* genes in the 440 microbial species that we regarded as putative ectoine producers from our database analysis (Fig. 1). In the vast majority (85%), the *ectABC* genes were located next to each other, strongly suggesting that their transcriptional organization is centrally based on an evolutionarily highly conserved operon structure (Fig. 6A). The basic *ectABC* gene cluster is frequently associated either with *ectD*, *ask_ect,* or *ectR,* and various genetic configurations of these *ect*-associated genes can be found in microbial genomes (Fig. 6A). The *ectD* gene may either be part of the *ectABC* operon or form a separate transcription unit somewhere else in the genome.

Our database analysis shows that the genetic organization of the *ect* gene cluster is well preserved in groups of microorganisms that are widely separated taxonomically (Fig. 6A). Nevertheless, there is a substantial sub-group (about 15%) of the putative ectoine/hydroxyectoine producers where the ectoine/hydroxyectoine biosynthetic genes and the functionally associated *ask_ect* and *ectR* genes are not organized in the well-defined gene clusters found in 85% of our reference data set. In this group of bacteria, the order of the various *ect* genes have either been scrambled or they have been separated from each other on the chromosome (Fig. 6B). It is currently unclear whether this non-canonical gene organization has any consequences for transcriptional induction or the level of ectoine/hydroxyectoine production in response to osmotic or temperature stress since, to the best of our knowledge, none of the putative ectoine/hydroxyectoine producers with deviating gene organizations have been functionally studied.

In a few of the hydroxyectoine producers (e.g., *Arthrobacter castelli* DSM 16402, *Marinobacter aquaeolei* VT8, *Rhodococcus opacus* B4, *Rhodococcus* sp. RHA1, *Chromohalobacter salexigens* DSM 3043), two copies of the *ectD* gene are found (Fig. 6B). Studies with *C. salexigens* DSM 3043 have shown that only one of these ectoine hydroxylases is responsible for the production of the majority of the hydroxyectoine found in this highly salt-tolerant bacterium [70]. In the genomes of other microorganisms, several *ectC*-type genes are found (e.g.; there are three *ectC* genes in *Marinobacter aquaeolei*). However, nothing is known whether these genes are all expressed and what (if any) the functional consequences of multiple, closely related EctC proteins within the same bacterium might be.

Another interesting finding of our database analysis is the identification of microorganisms that possess either two (e.g., *Phaeobacter arcticus* DSM 23566, *Vibrio cholerae* 0395, *Streptomyces flavogriseus* ATCC 33331) or even three (e.g., *Streptomyces clavuligerus* ATCC 27064) full copies of the ectoine/hydroxyectoine biosynthetic gene cluster. A pairwise comparison of the amino acid sequence of the various Ect proteins within a given species indicates that the increase in the *ect* gene copy number has likely arisen via gene duplication events since the corresponding proteins are all closely related to each other (data not shown). Whether these extra copies of the ectoine/hydroxyectoine biosynthetic genes are actually functionally expressed and whether the bacteria with the additional *ect* gene copies produce more ectoine than those with only one copy of the corresponding genes remains an interesting question for future studies.

### Concluding Remarks

Our comprehensive database analysis of finished microbial genomes revealed at the time of the search (July 2013) the presence of ectoine biosynthetic genes [19] in about 7% of the represented microorganisms, and about two thirds of these are predicted to produce hydroxyectoine [20], [27], [31] as well. Since hydroxyectoine often possess function-preserving and stress-relieving properties superior to those of ectoine [29], [35]–[38], one wonders why not all ectoine producers synthesize 5-hydroxyectoine since this can readily be accomplished from ectoine in a single step [20]. Part of the answer to this question becomes apparent when one considers the physiological requirements and the oxygen dependence of the ectoine/hydroxyectoine producer microorganisms. The EctD-catalyzed formation of 5-hydroxyectoine is an O_2_-dependent enzyme reaction [20], [43] and consequently none of the predicted hydroxyectoine producers is an obligate anaerobe, whereas both aerobic and anaerobic microorganisms can produce ectoine (Fig. 1).

Our data view the potential ectoine and hydroxyectoine producers within a wider taxonomic context (Fig. 1 and Fig. S8). With a few notable exceptions that revealed ectoine/hydroxyectoine biosynthetic genes in five members of the *Archaea* (*Methanosaeta* and *Nitrosopumilus* species), ectoine and hydroxyectoine producers are taxonomically affiliated with the domain of the *Bacteria*. We assessed the genetic organization of the ectoine/hydroxyectoine biosynthetic genes (Fig. 6) and those of proteins that are functionally associated with ectoine production, the specialized aspartokinase Ask_Ect [22], [45] and the transcriptional regulator EctR [25] (Fig. 1). By analyzing the occurrence of these proteins on a genome-wide scale and by viewing them in a taxonomic context, we derived the currently most comprehensive *in silico* analysis of the production potential for the stress protectants and chemical chaperones ectoine and 5-hydroxyectoine in microorganisms (Fig. 1 and 6; Fig. S8). This dataset can therefore serve as a solid benchmark for future assessments as microbial genome and metagenomic sequence analysis continues to progress in a rapid pace.

Our analysis of the genetic organization of the *ectABC/D* biosynthetic genes revealed a robust arrangement into an operon-like structure in taxonomically widely separated microorganisms (Fig. 6). This assessment does not only provide clues for their potential transcriptional organization, but also gives hints about which of these gene clusters might be useful as building blocks for synthetic ectoine/hydroxyectoine production in heterologous host systems [22], [56], [71]–[74]. For instance, we surmise that the ectoine/hydroxyectoine biosynthetic genes from *Kytococcus sedentarius* [75] might be effectively exploited as a synthetic “bio-brick” for this purpose. In this microorganism, the genes for both enzymes (Ask_Ect and Asd) required for the synthesis of the direct ectoine precursor, L-aspartate-β-semialdehyde [17], [18], [65], seem to be co-transcribed with the *ectABCD* operon (Fig. 6B). Co-expression of the *ask_ect-asd-ectABCD* gene cluster should help to avoid the build-up of potential bottlenecks during heterologous ectoine/hydroxyectoine production for biotechnological and medical purposes [15], [32], [33].

We placed special emphasis in our study on the further biochemical [20], [29] and structural analysis [44] of the ectoine hydroxylase. In terms of the EctD crystal structure, our new data reveal that the apo- and iron-liganded forms are virtually identical (Fig. 5A). Hence, the ectoine hydroxylase is pre-set in a configuration ready to accept the iron molecule (Fig. 5B) and the binding of the iron catalyst does not trigger large conformational changes. Together with the EctD proteins from *V. salexigens* and *S. coelicolor* that were previously studied biochemically [20], [29], the six ectoine hydroxylases examined here define the salient biochemical features (Table 1 and 2) of this group of closely related enzymes (Fig. S8). The ectoine hydroxylases analyzed so far all possess similar kinetic parameters and catalytic efficiencies (Table 1) [20], [29] but differ in their tolerance towards high temperature (Fig. 4) and in the influence of various salts on their enzyme activity (Table 2). It is hoped that the properties of some of the newly characterized EctD proteins will be suitable for further crystallographic studies so that a crystal structure of the ectoine hydroxylase with all its ligands (or its reaction product 5-hydroxyectoine) can be obtained in the future.

## Materials and Methods

### Chemicals

Ectoine and 5-hydroxyectoine were kind gifts from Dr. Thomas Lentzen and Dr. Irina Bagyan (bitop AG, Witten, Germany). 2-oxoglutarate (disodium salt) was obtained from Sigma-Aldrich (St. Louis, MO, USA). Anhydrotetracycline-hydrochloride (AHT), desthiobiotin, and Strep-Tactin Superflow chromatography material were purchased from IBA GmbH (Göttingen, Germany). X-Gal was obtained from AppliChem (Darmstadt, Germany), and the antibiotics kanamycin and ampicillin were purchased from Serva Electrophoresis GmbH (Heidelberg, Germany) and Carl Roth GmbH (Karlsruhe, Germany).

### Bacteria, Media and Growth Conditions

The *Escherichia coli* strain DH5α (Invitrogen, Karlsruhe, Germany) was used as host for recombinant plasmids and as overproduction strain for EctD-proteins; it was maintained routinely on LB agar plates and liquid media [76]. When it contained recombinant plasmids, either ampicillin (100 μg ml^−1^) or kanamycin (50 μg ml^−1^) was added to the growth medium to select for the presence of the plasmids. When appropriate, X-gal was included in agar plates to screen for the insertion of the desired DNA fragments into the cloning vector pENTRY-IBA20 (IBA, Göttingen, Gemany). For the overproduction of EctD-type proteins, minimal medium A (MMA) [76] was used that was supplemented with 0.5% (w/v) glucose as the carbon source, 0.5% (w/v) casaminoacids, 1 mM MgSO_4_, and 3 mM thiamine.

### Recombinant DNA Techniques and Construction of Plasmids

All recombinant DNA techniques followed routine procedures. To construct expression plasmids carrying either the *H. elongata* or the *S. alaskensis ectD* gene with a C-terminal *Strep*-tag-II affinity peptide, we amplified these *ectD* genes from chromosomal DNA with PCR using custom synthesized DNA primers. A BsaI restriction site was introduced at both ends of the amplified DNA fragments allowing the directed insertion of the PCR products into the expression vector pASK-IBA3 (IBA, Göttingen, Gemany) via BsaI restriction and ligation reactions. The generated plasmids were pMP32 (*ectD* from *H. elongata*) and pMP40 (*ectD* from *S. alaskensis*). Expression plasmids carrying the *P. stutzeri*, *P. lautus*, *A. ehrlichii* or *A. cryptum ectD* gene with a C-terminal *Strep*-tag-II affinity peptide were constructed using the IBA Stargate cloning system (IBA, Göttingen, Gemany). The *ectD* gene from *P. stutzeri* was amplified from chromosomal DNA via PCR using custom synthesized primers that carried synthetically added LguI DNA restriction sites at their ends; this PCR fragment was cloned into the donor vector pENTRY-IBA20 via LguI restriction and concurrent ligation thereby yielding plasmid pMP34. DNA sequences from *P. lautus*, *A. ehrlichii* and *A. cryptum* genes were retrieved from the database and this information was used for codon-optimized synthesis of *ectD* genes (GeneScript, Piscataway, USA). An LguI restriction site was added to both ends of these genes, and they were inserted into the pENTRY-IBA20 donor vector via LguI restriction and concurrent ligation. This generated plasmids pMP36 (*ectD* from *P. lautus*), pMP37 (*ectD* from *A. ehrlichii*), and pMP38 (*ectD* from *A. cryptum*). The synthetically manufactured *ectD* genes optimized for the expression in *E.coli* by GeneScript were deposited into the NCBI database with accession numbers JN019032 (*P. lautus ectD*), JN019031 (*A. ehrlichii ectD*) and JN019030 (*A. cryptum ectD*), respectively. To clone the *ectD* genes present on pMP34, pMP36, pMP37 and pMP38 into the pASG-IBA3 expression vector, Esp3I restriction and concurrent ligation of these plasmids and the expression vector pASG-IBA3 were carried out. In this way, in each of the recombinant *ectD* genes a short DNA fragment encoding the *Strep*-tag-II affinity peptide was added at their 3′-ends. The resulting plasmids were pMP41 (*ectD* from *P. stutzeri*), pMP43 (*ectD* from *P. lautus*), pMP44 (*ectD* from *A. ehrlichii*) and pMP48 (*ectD* from *A. cryptum*). The correct nucleotide sequence of all constructed plasmids was ascertained by DNA sequence analysis, which was carried out by Eurofins MWG Operon (Ebersberg, Germany).

### Overproduction and Purification of Recombinant EctD Enzymes

In each of the constructed recombinant plasmids, the *ectD* gene is expressed from the *tet* promoter under the control of the AHT inducible TetR repressor (encoded by the *tetR* gene present on the expression vector). Overproduction of the different ectoine hydroxylases was performed in a chemically defined medium containing glucose as the carbon source essentially as previously described [43], [44]. Briefly, cells of the *E. coli* strain DH5α harboring an appropriate plasmid were grown to an OD_578_ of about 0.7 at 37°C, the inducer AHT was then added to the culture to a final concentration of 0.2 mg mL^−1^, and the growth temperature was then reduced to 35°C; growth of the cultures was continued for two hours. The cells were harvested by centrifugation (10 min, 5000 rpm, 4°C) and stored at −20°C until further used. A Strep-Tactin Superflow column was used to purify the recombinant EctD enzymes by affinity chromatography as detailed previously [20], [43], [44]. The purified EctD proteins were shock-frozen in liquid nitrogen and stored at −80°C until they were further used in HPLC-based enzyme activity assays. These EctD preparations typically contained between 0.87 and 0.96 mole iron per mol of EctD protein.

To provide large amounts of EctD protein for the crystallization trials, the above described overexpression protocol was varied somewhat. Cells of *E. coli* DH5α harboring a recombinant plasmid carrying an *ectD* gene were grown at 37°C to an OD_578_ of about 0.5 in a flask set on an aerial shaker (180 rpm). The cultivation temperature was then reduced to 30°C, and the shaker speed were decreased to 100 rpm. The cells were then grown to an OD_578_ of about 0.7, after which the inducer (AHT) of the TetR repressor was added to the cultures at a final concentration of 0.2 mg mL^−1^. Cultures were grown for additional 2 hours and then harvested by centrifugation. By this modified overexpression protocol, the amount of purified EctD protein was increased from an average of 20–25 mg L^−1^ obtained by the initial protocol to 200–300 mg L^−1^. The purity of the EctD was assessed by SDS-PAGE (12% polyacrylamide) and concentrated by ultra-filtration on spin columns (Sartorius Stedim Biotech GmBH, Göttingen, Germany) to about 10 mg ml^−1^ prior to the crystallization experiments.

Gel filtration chromatography was performed to determine the size of each individual purified EctD protein by loading 1 ml of each protein solution [5 mg ml^−1^] onto a HiLoad 16/600 Superdex 200 pg column (GE Healthcare Europe GmbH, Freiburg, Germany) connected to an ÄKTA pure 25 L system (GE Healthcare Europe GmbH, Freiburg, Germany). The column was equilibrated and run in a 20 mM TES-buffer containing 150 NaCl. The evaluation of the column run was carried out with the Unicorn 6.3 software package (GE Healthcare Europe GmbH, Freiburg, Germany). A protein solution [3 mg ml^−1^] of carbonic anhydrase (from bovine erythrocytes) (29 kDa), albumin (from bovine serum) (66 kDa), and alcohol dehydrogenase (from *Saccharomyces cerevisiae*) (150 kDa) was used as standard (Gel Filtration Markers Kit; Sigma-Aldrich, St. Louis, MO, USA).

### Ectoine Hydroxylase Activity Assays

After affinity purification on Strep-Tactin Superflow material, the iron contents of the recombinantly produced EctD proteins were determined as described [77]. The hydroxylation of ectoine by EctD-type enzymes was measured by an HPLC-based enzyme assay [20]. In general, 30-μl reaction volumes containing 10 mM TES (pH 7.5), 1 mM FeSO_4_, 10 mM 2-oxoglutarate, 6 mM ectoine, and various amounts of the purified EctD enzymes were incubated aerobically in an Eppendorf thermo-mixer (Hamburg, Germany) (set to 700 rpm) at 32°C for 20 min. The enzyme reaction was stopped by adding 30-μl acetonitrile (100%) to the reaction mixture, immediately followed by centrifugation (10 min, 4°C, 32000×*g*) to remove the denatured EctD protein. The conversion of ectoine to 5-hydroxyectoine was assessed by loading 20 μl of the reaction mixture supernatant onto a GROM-SIL Amino-1PR column (125 mm by 4 mm; 3 μm particle size (GROM, Rottenburg-Hailfingen, Germany) attached to a UV-visible detector system (LINEAR UVIS 205; SYKAM, Fürstenfeldbruck, Germany) in an HPLC system (SYKAM). The absorbance of ectoine and 5-hydroxyectoine was monitored at 210 nm [11], [20], and the amount of 5-hydroxyectoine formed was quantitated using the ChromStar 7.0 software package (SYKAM, Fürstenfeldbruck, Germany).

To determine the biochemical properties of the different EctD-type proteins, the above-described standard enzyme assay was modified with respect to the incubation temperature, the buffer and pH conditions, and the salt content of the assay solution. To determine the kinetic parameters of the studied ectoine hydroxylases, each of the different EctD enzymes was assayed at its optimal conditions (Table 1) with varied concentrations of either ectoine (between 0 and 80 mM) or 2-oxoglutarate (between 0 and 50 mM). To assess the ability of the EctD protein to perform the reverse enzyme reaction (forming ectoine from 5-hydroxyectoine), samples of the purified *V. salexigens* EctD protein were incubated under the assay conditions described above, except that various concentrations of 5-hydroxyectoine (from 6 mM to 100 mM) instead of ectoine, succinate (from 5 to 40 mM) instead of 2-oxoglutarate, and bicarbonate (between 5 mM to 20 mM) were used. These reaction samples were incubated (either with or without shaking in a thermo-mixer) for various time periods (from 20 min to 24 hours), and processed as described above. The products of the enzyme reactions were then analyzed by HPLC [20].

### Database Searches, Alignments of Amino Acid Sequences, and Construction of Phylogenetic Trees of EctC- and EctD-type Proteins

The bioinformatics tools available at the DOE Joint Genome Institute website (http://www.jgi.doe.gov) [46] were used to retrieve EctC- and EctD-type protein sequences from finished microbial genomes (search date: 07/31/2013). For these database searches, the amino acid sequences of the EctC (accession number: AAY29688) and EctD (accession number: AAY29689) proteins from *V. salexigens* [20] were used as the query sequence using the BLAST program [78]. The retrieved EctC and EctD protein sequences were aligned and compared using ClustalW [62]. Based on these alignments, phylogenetic trees were calculated using the iTOL-software package (http://itol.embl.de/) [61] to visualize the distribution of EctC and EctD proteins among members of the *Bacteria* and *Archaea*. The genetic organization of the *ectABC/*(*ectD*) gene cluster and its flanking sequences were analyzed using the online tool available from the DOE Joint Genome Institute website [46].

### Crystallization of the *V. Salexigens* EctD Protein in its Iron-free Form

Crystallization trials were performed using the sitting-drop vapor diffusion method at 20°C. A homogenous protein solution of the affinity-purified EctD protein (in 20 mM TES pH 7.5, 80 mM NaCl) was concentrated to 10 mg/ml prior to crystallization experiments. EctD crystals were grown by mixing 1.5 μl protein solution with 1.5 μl reservoir solution containing 100 mM MES pH 5.0 and 1.2 M ammonium sulfate; the EctD crystals grew within 6–12 days to their final size of around 80×90×100 μm^3^. Crystals were cryoprotected by carefully adding 1 μl 100% glycerol to the crystallization drop before freezing the crystals in liquid nitrogen.

### Data Collection, Refinement and Crystallographic Analysis of the EctD Protein

EctD crystals diffracted X-rays to a minimum resolution of 1.85 Å for the apo-EctD. The dataset was collected at the ID23-EH2 beamline at the ESRF (Grenoble, France) and processed with XDS [79]. The crystal structure of the iron-bound *V. salexigens* EctD protein (PDB code: 3EMR) [44] was used as a template to obtain initial phases using PHASER [80]. The structure was further refined using REFMAC5 [81] and manually adjusted using COOT [82]. Dataset and refinement statistics for the apo-EctD crystal structure are listed in Table S1 and were analyzed with Procheck [83]. The crystallographic information for the *V. salexigens* apo-EctD protein was deposited in the Protein Data Base (PDB) [84] with the PDB accession code 4NMI. Figures of protein molecules derived from crystal structures were prepared using the PyMol software suit (www.pymol.org).

## Supporting Information

Figure S1 Biochemical properties of the EctD enzyme from *Halomonas elongata*. The enzyme activity of the ectoine hydroxylase from *H. elongata* is shown with respect to (A) the temperature optimum, (B) the pH optimum, and the influence of different salts: (C) potassium chloride, (D) sodium chloride, (E) potassium glutamate, and (F) ammonium chloride.
(TIF)

Figure S2 Biochemical properties of the EctD enzyme from *Pseudomonas stutzeri*. The enzyme activity of the ectoine hydroxylase from *P. stutzeri* is shown with respect to (A) the temperature optimum, (B) the pH optimum, and the influence of different salts: (C) potassium chloride, (D) sodium chloride, (E) potassium glutamate, and (F) ammonium chloride.
(TIF)

Figure S3 Biochemical properties of the EctD enzyme from *Paenibacillus lautus*. The enzyme activity of the ectoine hydroxylase from *P. lautus* is shown with respect to (A) the temperature optimum, (B) the pH optimum, and the influence of different salts: (C) potassium chloride, (D) sodium chloride, (E) potassium glutamate, and (F) ammonium chloride.
(TIF)

Figure S4 Biochemical properties of the EctD enzyme from *Alkalilimnicola ehrlichii*. The enzyme activity of the ectoine hydroxylase from *A. ehrlichii* is shown with respect to (A) the temperature optimum, (B) the pH optimum, and the influence of different salts: (C) potassium chloride, (D) sodium chloride, (E) potassium glutamate, and (F) ammonium chloride.
(TIF)

Figure S5 Biochemical properties of the EctD enzyme from *Acidiphilium cryptum*. The enzyme activity of the ectoine hydroxylase from *A. cryptum* is shown with respect to (A) the temperature optimum, (B) the pH optimum, and the influence of different salts: (C) potassium chloride, (D) sodium chloride, (E) potassium glutamate, and (F) ammonium chloride.
(TIF)

Figure S6 Gel filtration analysis of the *Sphingopyxis alaskensis* EctD protein. The *S. alaskensis* EctD protein was purified by affinity chromatography and its quaternary structure was then assessed by gel filtration analysis on a HiLoad 16/600 Superdex 200 pg column. The column was equilibrated and run in a 20 mM TES-buffer containing 150 NaCl. A protein solution [3 mg/ml] of carbonic anhydrase (from bovine erythrocytes) (29 kDa), albumin (from bovine serum) (66 kDa), and alcohol dehydrogenase (from *Saccharomyces cerevisiae*) (150 kDa) was used as a standard. The calculated molecular mass of the *S. alaskensis* EctD protein with the attached *Strep-*tag-II affinity peptide (nine amino acids) is 35.29 kDa; the molecular mass calculated from the column run was 70.38 kDa. Arrows indicate the elution of the standard proteins from the gel filtration column. mAU: milli absorption units.
(TIF)

Figure S7 Enzyme activity of the ectoine hydroxylase is not reversible. The forward and backward enzyme reactions of the EctD protein from *S. alaskensis* were tested, and the formation of ectoine and hydroxyectoine was monitored by HPLC analysis. (a) Chromatograms from HPLC measurements monitored at 210 nm of a mixture of commercially available ectoine and 5-hydroxyectoine standards. (b) HPLC tracing of the EctD-catalyzed enzyme reaction mixture that initially contained 6 mM ectoine; the enzyme assay was run for 20 min. (c) HPLC tracing of the EctD-catalyzed “reverse” enzyme reaction mixture that initially contained 6 mM hydroxyectoine; the enzyme assay was run for 24 h.
(TIF)

Figure S8 Phylogenetic tree of EctD-type proteins. The phylogenetic tree of ectoine hydroxylases shown is based on the alignment of EctD amino acid sequences identified by a BLAST search at the JGI Web-server, and that were then aligned using ClustalW. The phylogenetic distribution of the aligned EctD proteins was assessed via the iTOL Web-server. Evolutionary distances are not given. The color code indicates the distribution of EctD among members of the *Bacteria* and *Archaea*.
(TIF)

Table S1 Data collection and refinement statistics for the crystal structure of the EctD protein from *V. salexigens* in its iron-free form.
(DOC)
