# Supplementary figures and images for: Biochemical Properties of Ectoine Hydroxylases from Extremophiles and Their Wider Taxonomic Distribution among Microorganisms
Source: PLoS One. 2014 Apr 8;9(4):e93809. doi: 10.1371/journal.pone.0093809 (PMC3979721; doi:10.1371/journal.pone.0093809)

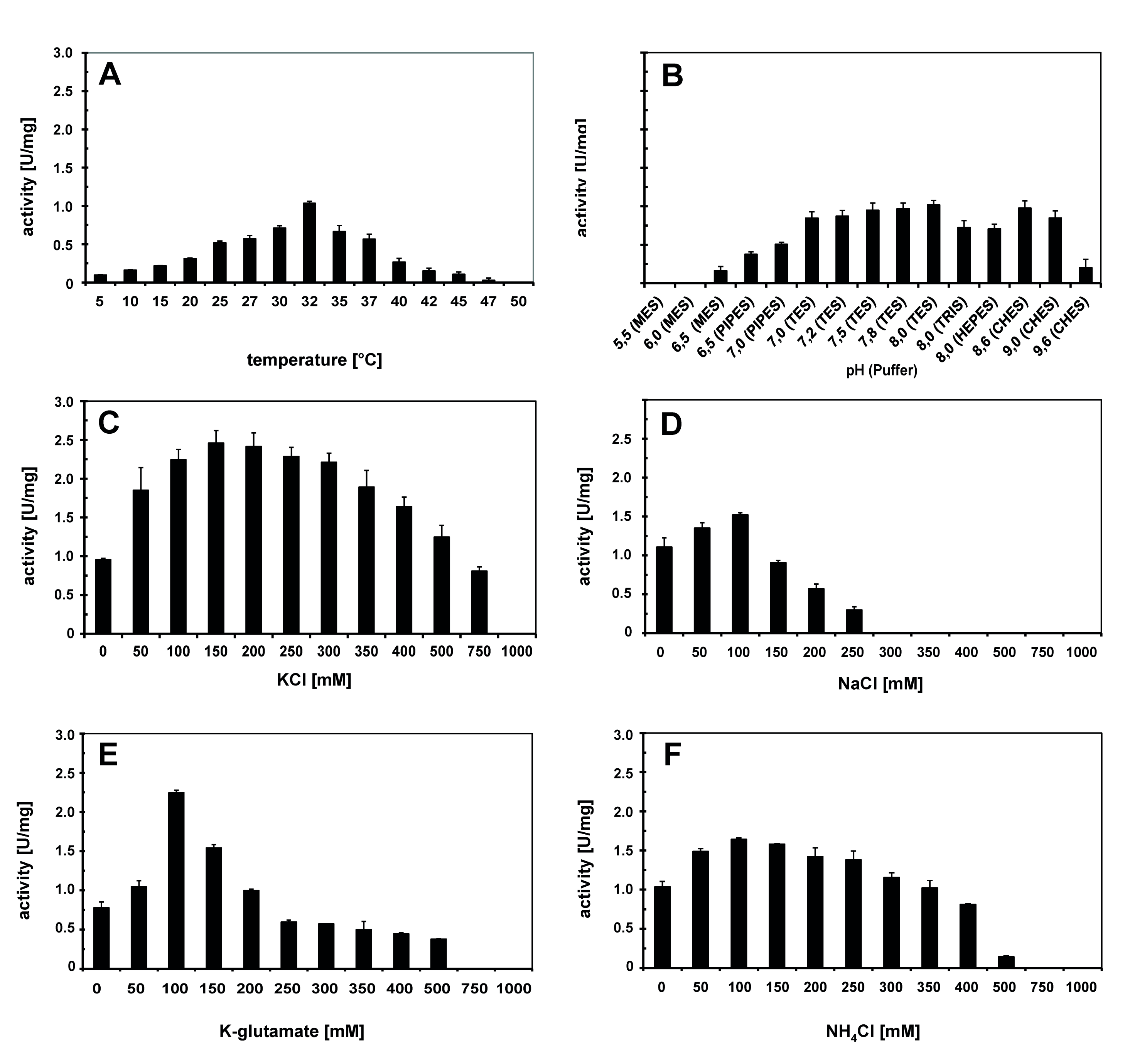

Supplement: Figure S1 — Biochemical properties of the EctD enzyme from Halomonas elongata. The enzyme activity of the ectoine hydroxylase from H. elongata is shown with respect to (A) the temperature optimum, (B) the pH optimum, and the influence of different salts: (C) potassium chloride, (D) sodium chloride, (E) potassium glutamate, and (F) ammonium chloride. (TIF) [file pone.0093809.s001.tif]

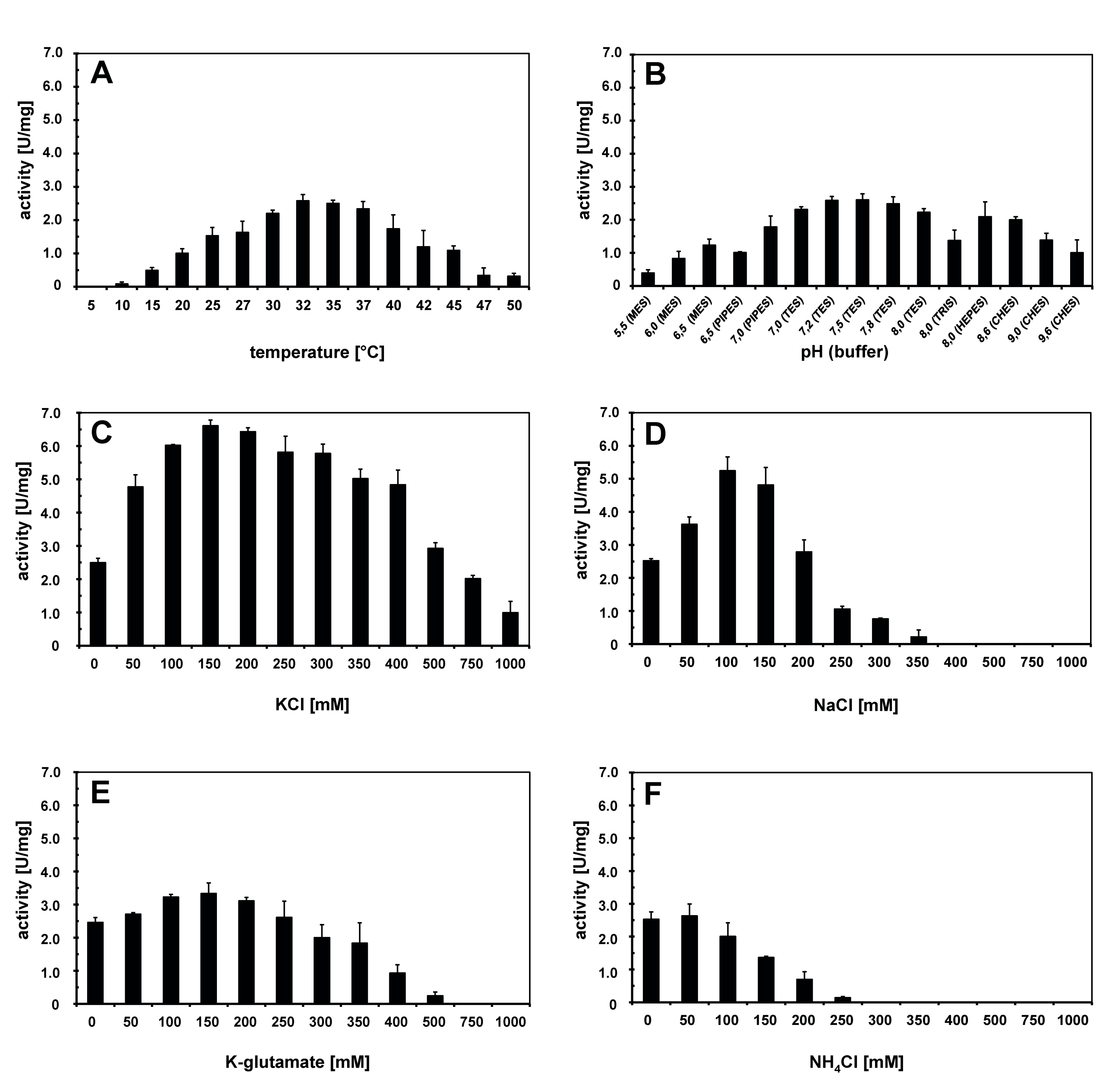

Supplement: Figure S2 — Biochemical properties of the EctD enzyme from Pseudomonas stutzeri. The enzyme activity of the ectoine hydroxylase from P. stutzeri is shown with respect to (A) the temperature optimum, (B) the pH optimum, and the influence of different salts: (C) potassium chloride, (D) sodium chloride, (E) potassium glutamate, and (F) ammonium chloride. (TIF) [file pone.0093809.s002.tif]

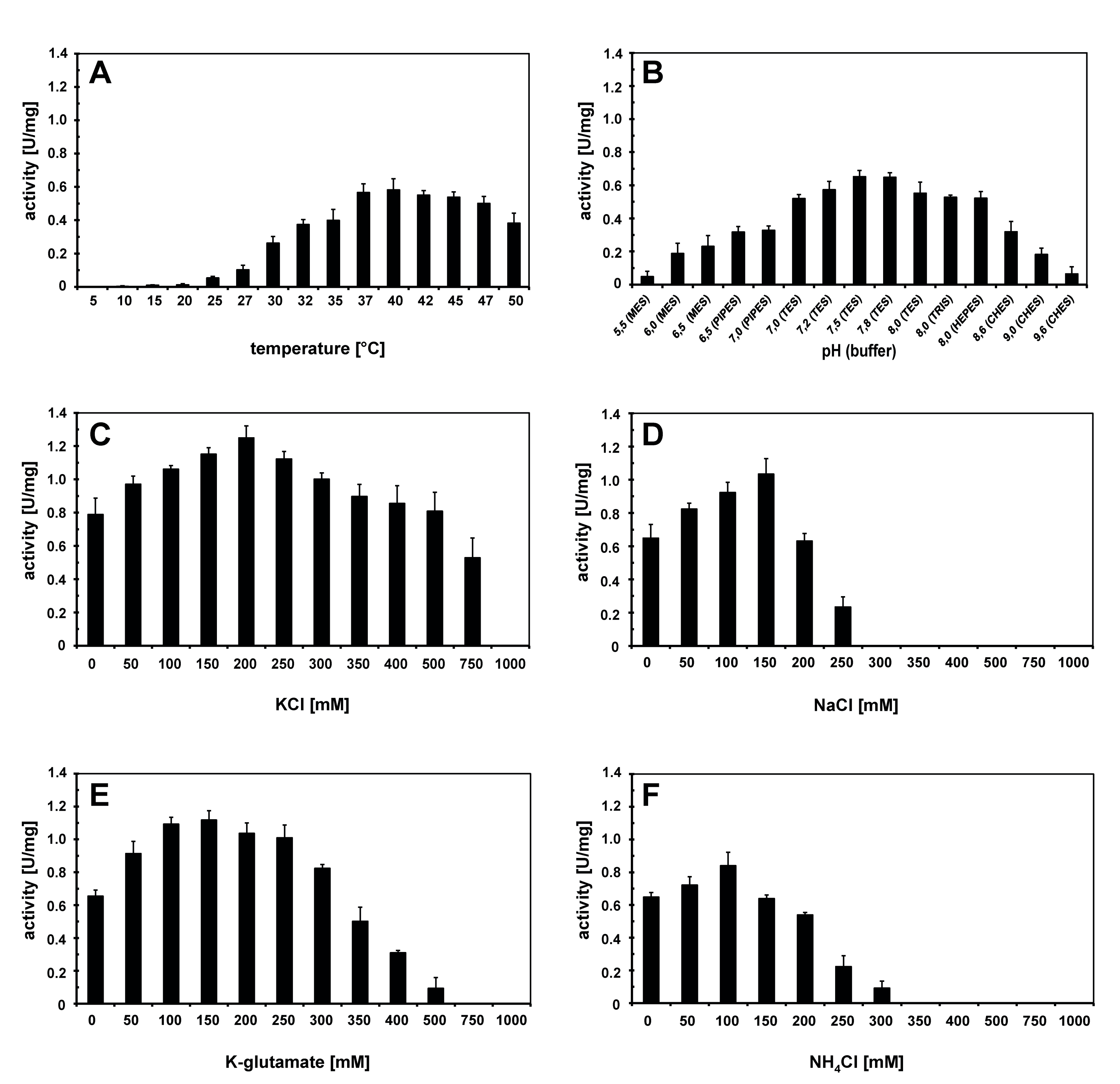

Supplement: Figure S3 — Biochemical properties of the EctD enzyme from Paenibacillus lautus. The enzyme activity of the ectoine hydroxylase from P. lautus is shown with respect to (A) the temperature optimum, (B) the pH optimum, and the influence of different salts: (C) potassium chloride, (D) sodium chloride, (E) potassium glutamate, and (F) ammonium chloride. (TIF) [file pone.0093809.s003.tif]

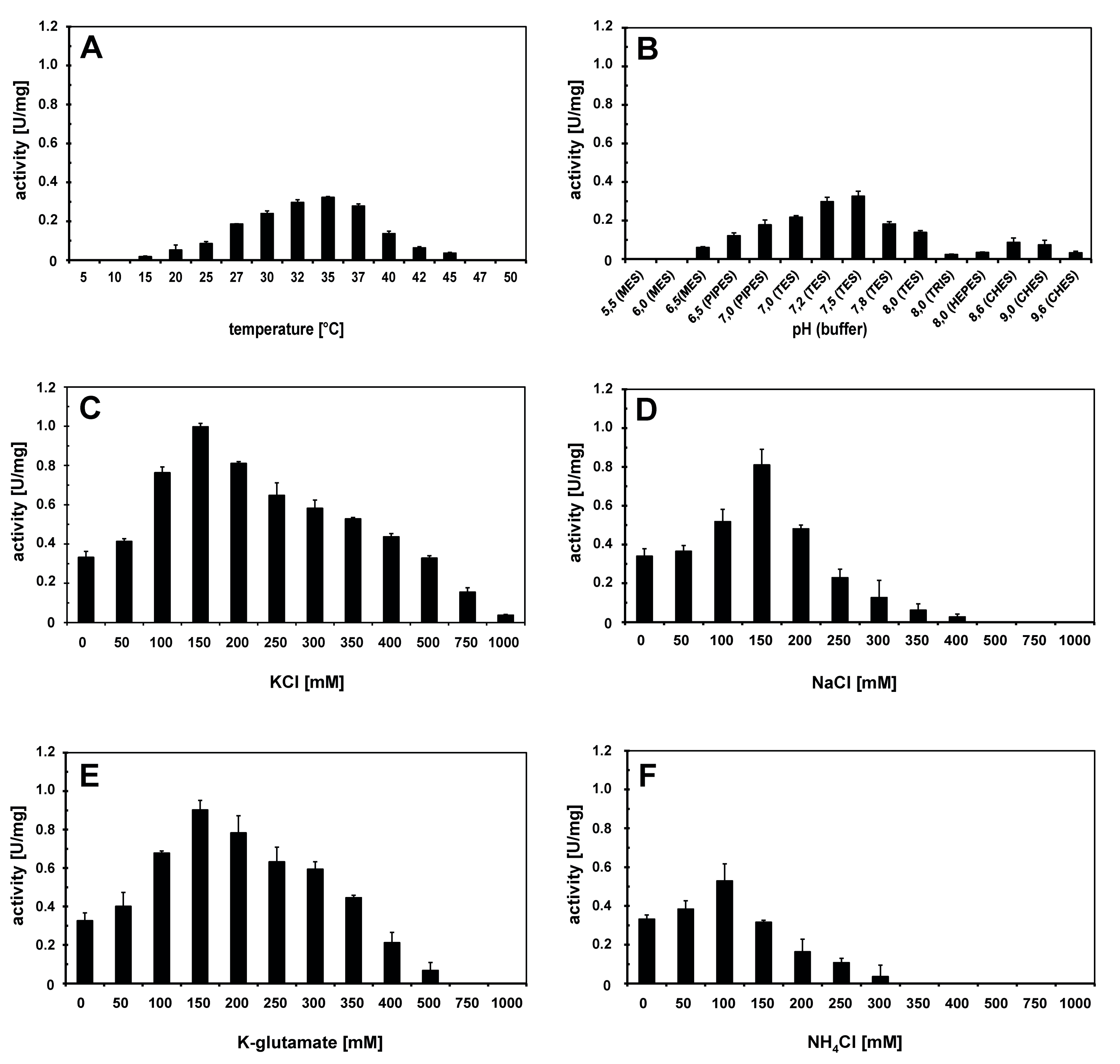

Supplement: Figure S4 — Biochemical properties of the EctD enzyme from Alkalilimnicola ehrlichii. The enzyme activity of the ectoine hydroxylase from A. ehrlichii is shown with respect to (A) the temperature optimum, (B) the pH optimum, and the influence of different salts: (C) potassium chloride, (D) sodium chloride, (E) potassium glutamate, and (F) ammonium chloride. (TIF) [file pone.0093809.s004.tif]

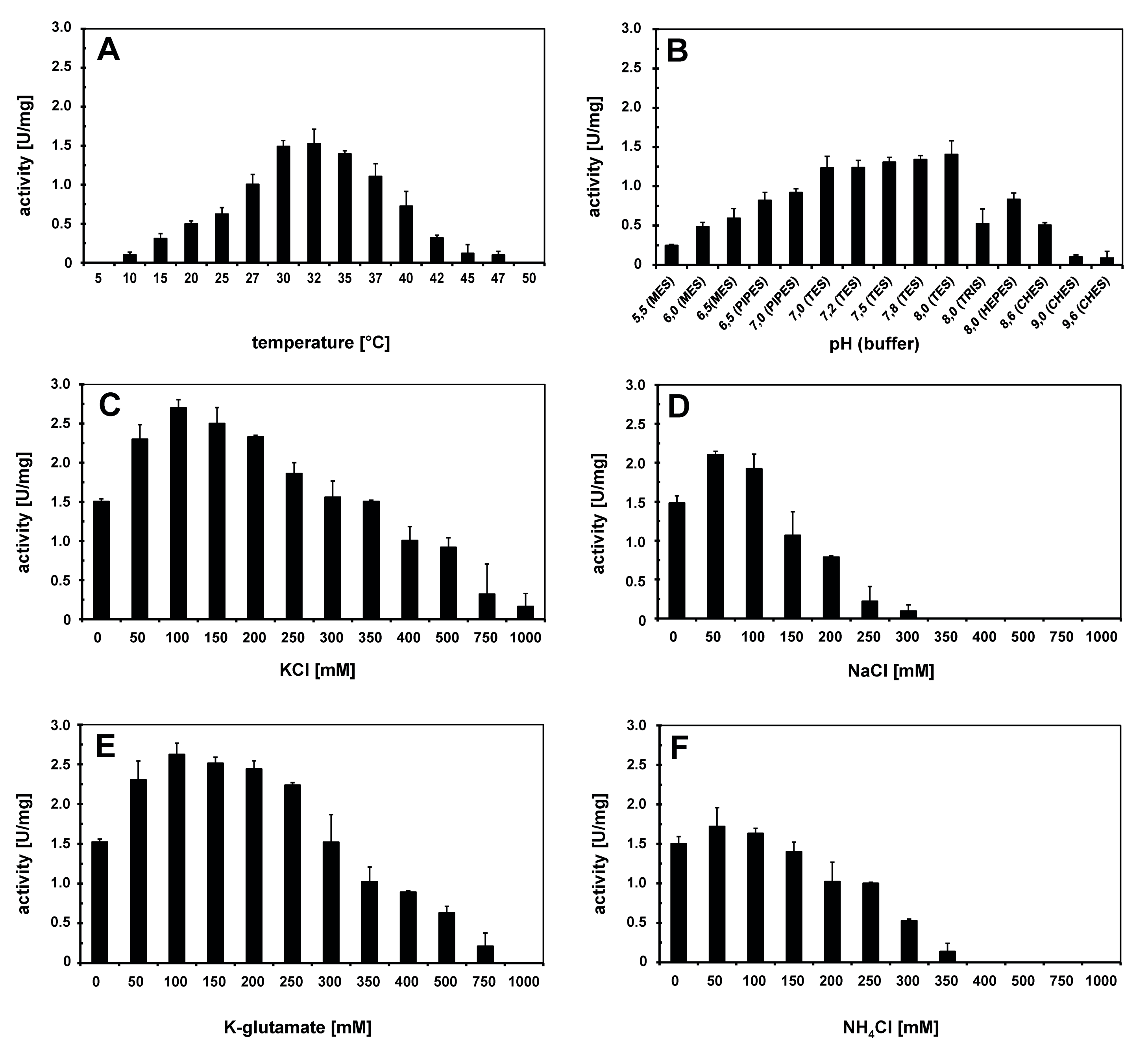

Supplement: Figure S5 — Biochemical properties of the EctD enzyme from Acidiphilium cryptum. The enzyme activity of the ectoine hydroxylase from A. cryptum is shown with respect to (A) the temperature optimum, (B) the pH optimum, and the influence of different salts: (C) potassium chloride, (D) sodium chloride, (E) potassium glutamate, and (F) ammonium chloride. (TIF) [file pone.0093809.s005.tif]

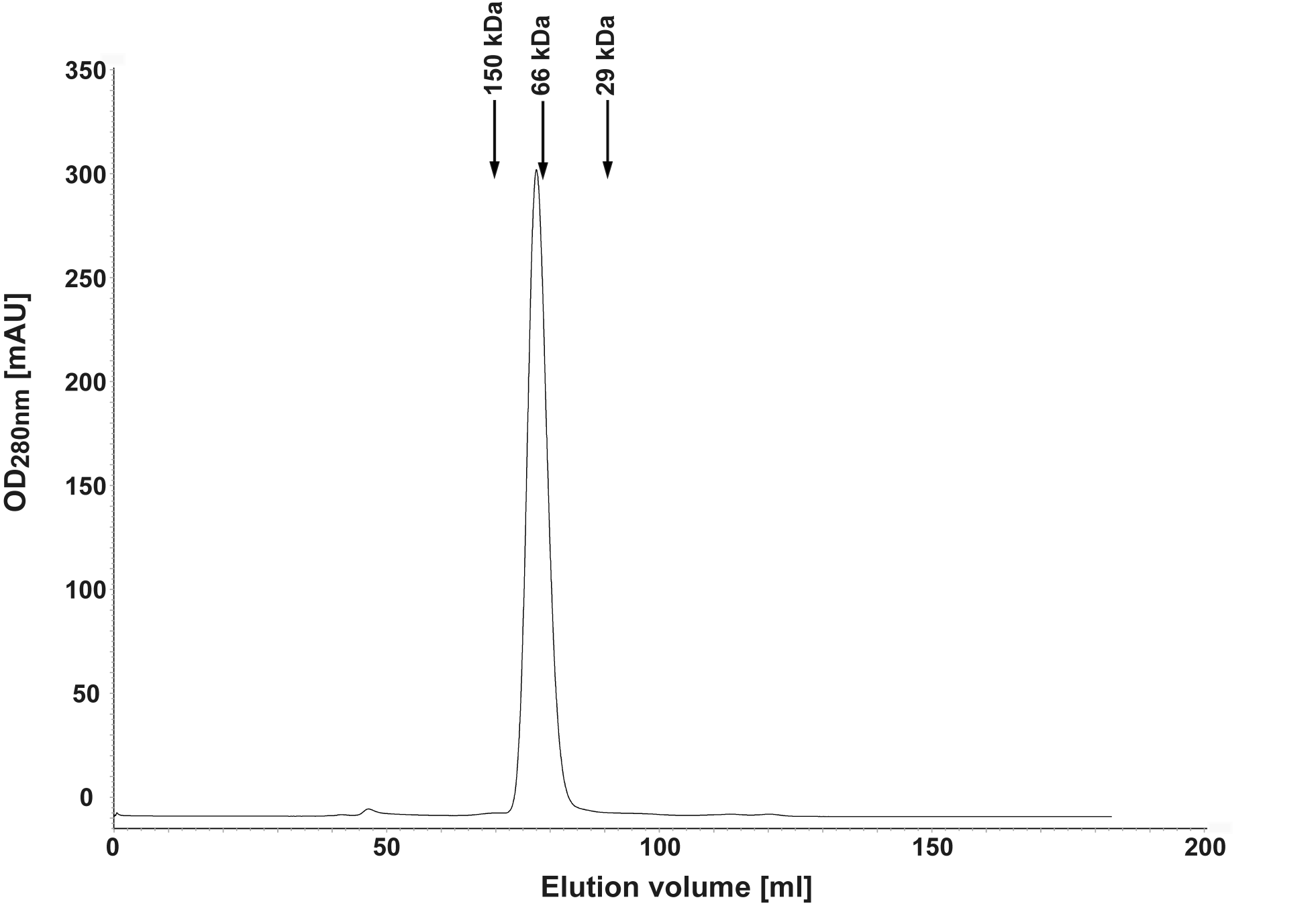

Supplement: Figure S6 — Gel filtration analysis of the Sphingopyxis alaskensis EctD protein. The S. alaskensis EctD protein was purified by affinity chromatography and its quaternary structure was then assessed by gel filtration analysis on a HiLoad 16/600 Superdex 200 pg column. The column was equilibrated and run in a 20 mM TES-buffer containing 150 NaCl. A protein solution [3 mg/ml] of carbonic anhydrase (from bovine erythrocytes) (29 kDa), albumin (from bovine serum) (66 kDa), and alcohol dehydrogenase (from Saccharomyces cerevisiae) (150 kDa) was used as a standard. The calculated molecular mass of the S. alaskensis EctD protein with the attached Strep-tag-II affinity peptide (nine amino acids) is 35.29 kDa; the molecular mass calculated from the column run was 70.38 kDa. Arrows indicate the elution of the standard proteins from the gel filtration column. mAU: milli absorption units. (TIF) [file pone.0093809.s006.tif]

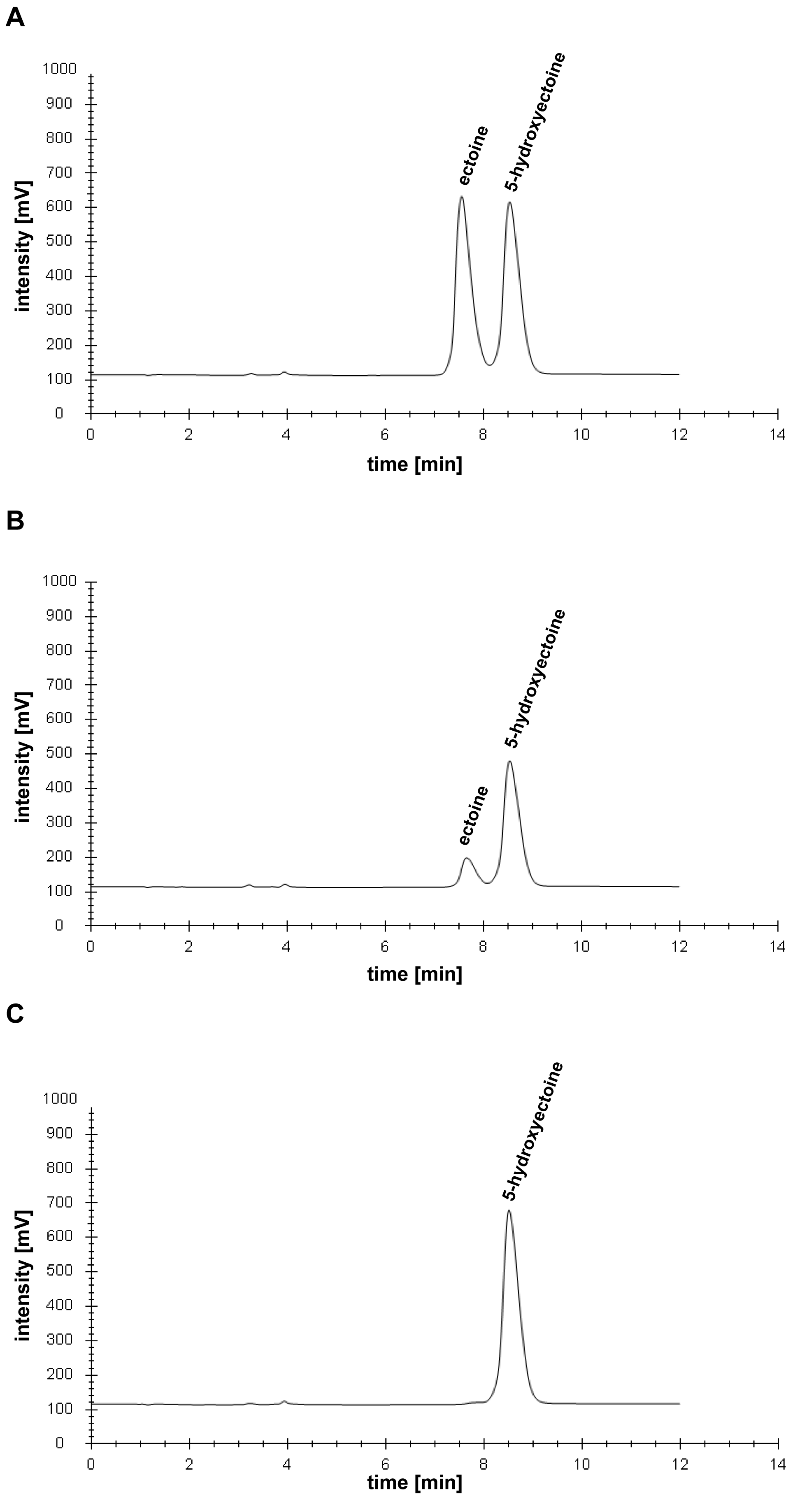

Supplement: Figure S7 — Enzyme activity of the ectoine hydroxylase is not reversible. The forward and backward enzyme reactions of the EctD protein from S. alaskensis were tested, and the formation of ectoine and hydroxyectoine was monitored by HPLC analysis. (a) Chromatograms from HPLC measurements monitored at 210 nm of a mixture of commercially available ectoine and 5-hydroxyectoine standards. (b) HPLC tracing of the EctD-catalyzed enzyme reaction mixture that initially contained 6 mM ectoine; the enzyme assay was run for 20 min. (c) HPLC tracing of the EctD-catalyzed “reverse” enzyme reaction mixture that initially contained 6 mM hydroxyectoine; the enzyme assay was run for 24 h. (TIF) [file pone.0093809.s007.tif]

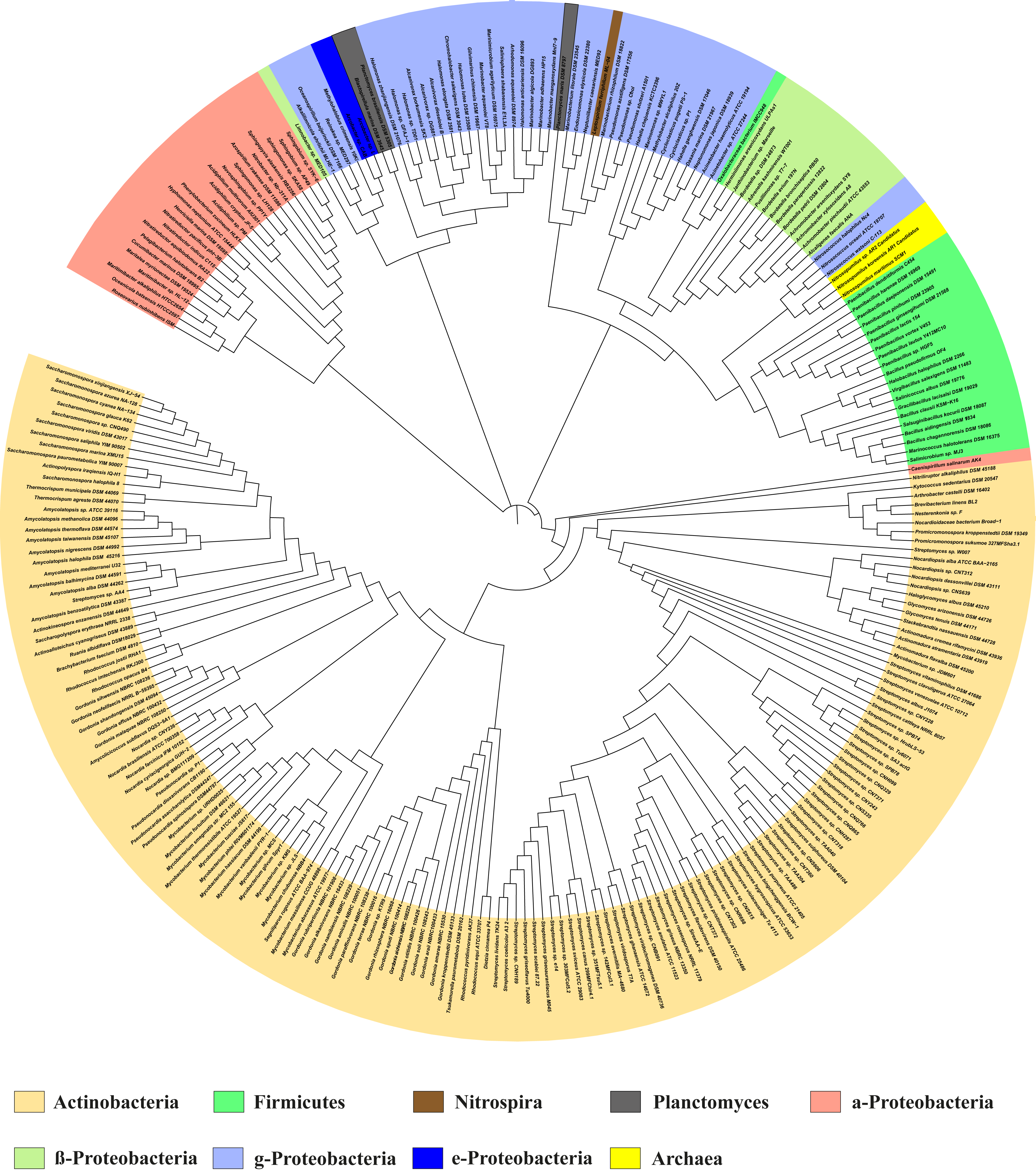

Supplement: Figure S8 — Phylogenetic tree of EctD-type proteins. The phylogenetic tree of ectoine hydroxylases shown is based on the alignment of EctD amino acid sequences identified by a BLAST search at the JGI Web-server, and that were then aligned using ClustalW. The phylogenetic distribution of the aligned EctD proteins was assessed via the iTOL Web-server. Evolutionary distances are not given. The color code indicates the distribution of EctD among members of the Bacteria and Archaea. (TIF) [file pone.0093809.s008.tif]
