# Supplementary material for: Biochemical Properties of Ectoine Hydroxylases from Extremophiles and Their Wider Taxonomic Distribution among Microorganisms
Source: PLoS One. 2014 Apr 8;9(4):e93809. doi: 10.1371/journal.pone.0093809 (PMC3979721; doi:10.1371/journal.pone.0093809)
Supplement: Table S1 — Data collection and refinement statistics for the crystal structure of the EctD protein from V. salexigens in its iron-free form. (DOC) [file pone.0093809.s009.doc]

**Table S1**

| **A. Crystal parameters at 100 K** |  |  |  |  |
| --- | --- | --- | --- | --- |
| Space group |  |  |  | P6_5_22 |
| Unit Cell parameters |  |  |  |  |
| a, b, c (Å) |  |  |  | 102.89 102.89 159.65 |
| α=β(°) |  |  |  | 90.0 |
| χ (°) |  |  |  | 120.0 |
|  |  |  |  |  |
| **B. Data collection and processing** |  |  |  |  |
| Wavelength (Å) |  |  |  | 0.87260 |
| Resolution (Å) |  |  |  | 30-1.9 (2.0-1.9) |
| Mean redundancy |  |  |  | 3.7 (3.0) |
| Unique reflections |  |  |  | 37155 |
| Completeness (%) |  |  |  | 92.8 (89.5) |
| I/σ |  |  |  | 16.4 (2.9) |
| R_sym_ |  |  |  | 5.5 (34.0) |
|  |  |  |  |  |
| **C. Refinement statistics** |  |  |  |  |
| R_F_ (%) |  |  |  | 17.6 |
| R_free_ (%) |  |  |  | 20.1 |
| rmsd from ideal |  |  |  |  |
| Bond lengths (Å) |  |  |  | 0.024 |
| Bond angles (deg.) |  |  |  | 1.126 |
| Average B-factors (Å^2^) |  |  |  | 43.1 |
| Ramachandran plot |  |  |  |  |
| Most favored (%) |  |  |  | 96.4 |
| Allowed (%) |  |  |  | 3.6 |
| Generously allowed (%) |  |  |  | 0 |
| Disallowed (%) |  |  |  | 0 |
|  |  |  |  |  |
| **D. Model content** |  |  |  |  |
| Monomers/ASU |  |  |  | 1 |
| Protein residues |  |  |  | 2-195 211-296 |
| Ligand |  |  |  | - |
| Water molecules |  |  |  | 340 |
